# *In Vitro* and *in Silico* Evidence of Phosphatase Diversity in the Biomineralizing Bacterium *Ramlibacter tataouinensis*

**DOI:** 10.3389/fmicb.2017.02592

**Published:** 2018-01-11

**Authors:** Fériel Skouri-Panet, Karim Benzerara, Julie Cosmidis, Céline Férard, Géraldine Caumes, Gilles De Luca, Thierry Heulin, Elodie Duprat

**Affiliations:** ^1^Centre National de la Recherche Scientifique, Institut de Minéralogie, de Physique des Matériaux et de Cosmochimie, Sorbonne Universités, UMR 7590, Muséum National d'Histoire Naturelle, Université Pierre et Marie Curie, IRD 206, Paris, France; ^2^Department of Geological Sciences, University of Colorado, Boulder, CO, United States; ^3^Laboratoire d'Écologie Microbienne de la Rhizosphère et Environnements Extrêmes, UMR 7265, Aix Marseille Univ, Centre National de la Recherche Scientifique, Commissariat à l'Énergie Atomique et aux Énergies Alternatives, Saint-Paul-lez-Durance, France

**Keywords:** microbial phosphatases, biomineralization, phosphatogenesis, metal-phosphate mineral phases, enzymatic activity, genomic repertoire, organic phosphates, hydroxyapatite

## Abstract

Microbial phosphatase activity can trigger the precipitation of metal-phosphate minerals, a process called phosphatogenesis with global geochemical and environmental implications. An increasing diversity of phosphatases expressed by diverse microorganisms has been evidenced in various environments. However, it is challenging to link the functional properties of genomic repertoires of phosphatases with the phosphatogenesis capabilities of microorganisms. Here, we studied the betaproteobacterium *Ramlibacter tataouinensis* (*Rta*), known to biomineralize Ca-phosphates in the environment and the laboratory. We investigated the functional repertoire of this biomineralization process at the cell, genome and molecular level. Based on a mineralization assay, *Rta* is shown to hydrolyse the phosphoester bonds of a wide range of organic P molecules. Accordingly, its genome has an unusually high diversity of phosphatases: five genes belonging to two non-homologous families, *phoD* and *phoX*, were detected. These genes showed diverse predicted cis-regulatory elements. Moreover, they encoded proteins with diverse structural properties according to molecular models. Heterologously expressed PhoD and PhoX in *Escherichia coli* had different profiles of substrate hydrolysis. As evidenced for *Rta* cells, recombinant *E. coli* cells induced the precipitation of Ca-phosphate mineral phases, identified as poorly crystalline hydroxyapatite. The phosphatase genomic repertoire of *Rta* (containing phosphatases of both the PhoD and PhoX families) was previously evidenced as prevalent in marine oligotrophic environments. Interestingly, the Tataouine sand from which *Rta* was isolated showed similar P-depleted, but Ca-rich conditions. Overall, the diversity of phosphatases in *Rta* allows the hydrolysis of a broad range of organic P substrates and therefore the release of orthophosphates (inorganic phosphate) under diverse trophic conditions. Since the release of orthophosphates is key to the achievement of high saturation levels with respect to hydroxyapatite and the induction of phosphatogenesis, *Rta* appears as a particularly efficient driver of this process as shown experimentally.

## Introduction

Phosphorus is essential to life but a limiting nutrient in many ecosystems (Paytan and McLaughlin, 2007; Pasek, 2008). In response, cellular adaptations have been selected in microorganisms to catalyze an efficient uptake and recycling of P (Sowell et al., 2008; Martiny et al., 2009; Coleman and Chisholm, 2010; Sebastián et al., 2012; Martin et al., 2014). Under certain conditions, the microbial release of phosphorus generates locally high P concentrations, sometimes triggering the precipitation of metal-phosphate phases, a process called phosphatogenesis (Föllmi, 1996; Arning et al., 2009; Goldhammer et al., 2010; Cosmidis et al., 2014). The implication of bacteria in the biomineralization of metal-phosphates, including calcium-, iron- or uranium-phosphates has been suggested based on (1) the frequent occurrence of bacteria fossil in phosphate-rich sedimentary deposits (Zanin and Zamirailova, 2011; Bailey et al., 2013; Cosmidis et al., 2013a,b) and (2) laboratory biomineralization experiments (Powers et al., 2002; Templeton et al., 2003; Beazley et al., 2009; Miot et al., 2009; Rivadeneyra et al., 2010; Yung and Jiao, 2014; Cosmidis et al., 2015). Deciphering the microbial processes involved will help designing better models for the formation of sedimentary phosphate minerals, a major sink in the global cycle of P (Föllmi, 1996), as well as developing new bioremediation strategies to sequester metal pollutants (e.g., Martinez et al., 2007; Handley-Sidhu et al., 2010; Mondani et al., 2011; Liang et al., 2016).

Several studies suggest that microbial phosphatases are particularly important in phosphatogenesis (Hirschler et al., 1990; Blake et al., 1998; Macaskie et al., 2000; Beazley et al., 2007; Nilgiriwala et al., 2008; Shelobolina et al., 2009). Phosphatases catalyse the hydrolysis of phosphoesters, an important reservoir of P in many environments (Clark et al., 1998; Young and Ingall, 2010). This hydrolysis releases orthophosphates, favoring mineral precipitation in the presence of cations such as Ca^2+^, Fe^2+^, Fe^3+^, or U^6+^ (Powers et al., 2002; Cosmidis et al., 2015). Several types of phosphatases are known, including the alkaline phosphatase superfamily (Coleman, 1992) composed of at least 3 non-homologous families named PhoA, PhoD, and PhoX (Kim and Wyckoff, 1991; Rodriguez et al., 2014; Yong et al., 2014). Interestingly, this superfamily exhibits a broad diversity in (1) substrate specificity profile: for example, some proteins hydrolyze phosphate monoesters such as glycerophosphate or phosphoproteins, while other are more efficient at hydrolyzing phosphate diesters such as nucleic acids; (2) cofactors such as Zn^2+^, Ca^2+^, Mg^2+^, Fe^3+^; (3) cellular location; and (4) regulation pathways (Eder et al., 1996; Wojciechowski et al., 2002; Zalatan et al., 2006; Zaheer et al., 2009). Finally, the environmental distribution of these families differs, suggesting an adaptive strategy of microbial communities in response to multiple environmental drivers (Ragot et al., 2017), e.g., the relative availability of inorganic phosphorus and metal co-factors (Kathuria and Martiny, 2011; Moore, 2014). For example, PhoX and PhoD families are more abundant than PhoA under marine Zn- and P-depleted conditions (Luo et al., 2009; Martiny et al., 2009; Sebastian and Ammerman, 2009; Temperton et al., 2011). Yet, we have a poor understanding on how this diversity (at cell or community level) controls the capability of ecosystems to induce Ca-phosphate biomineralization. It can be hypothesized that in a given environment where a certain type of organic P substrate prevails and/or some metal co-factors are limiting, some phosphatases might be more efficient than others at releasing orthophosphates and therefore inducing phosphatogenesis. With the possibility to access the whole genomes of numerous microorganisms, an outstanding question to address is whether a microbe's phosphatase gene content can help predict its mineralization capabilities under particular conditions.

In this context, we studied *Ramlibacter tataouinensis* strain TTB310 (*Rta*), a betaproteobacterium isolated from a semi-arid region in Tunisia, where abundant calcification occurs (Gillet et al., 2000; Benzerara et al., 2003; Heulin et al., 2003). *Rta* is able to precipitate calcium phosphates *in vitro* (Benzerara et al., 2004). Although the involvement of a phosphatase was speculated, the molecular mechanisms inducing biomineralization by *Rta* remain unknown. The genome of the strain has been sequenced and annotated (De Luca et al., 2011), offering the possibility to explore the functional repertoire of *Rta* phosphatases. Here, we combined bioinformatics, molecular biology, biochemistry and mineralogy to characterize *Ramlibacter tataouinensis* phosphatase properties in relation with its capability to induce phosphatogenesis.

## Materials and methods

### Chemicals

All reactants, including *p*-nitrophenylphosphate (*p*NPP), bis *p*-nitrophenylphosphate (bis-*p*NPP), Thymidine 5′-monophosphate *p*-nitrophenylester sodium salt (T*p*NPP), calcium glycerophosphate (CaGP) and sodium glycerophosphate (NaGP) were purchased from Sigma-Aldrich. *p*NPP and glycerophosphate (GP) are phosphomonoesters while bis-*p*NPP and T*p*NPP are phosphodiesters.

### *Rta* cultivation

*Ramlibacter tataouinensis* strain TTB310 (*Rta*) was described by Heulin et al. (2003) and its genome analyzed by De Luca et al. (2011). Here, *Rta* was cultivated in fivefold diluted LB medium (LB) at 30°C with orbital shaking (100 rpm) in the dark. The stationary phase (cell density between 10^7^ and 10^8^ cells/ml) was achieved after 4 weeks.

### Gene cloning and recombinant expression of phosphatase genes in *E. coli*

Phosphatase genes predicted from *Rta* genome (see below for details on the functional annotation procedure) were recombinantly expressed in *E. coli* to characterize some properties of these enzymes. Sequences were optimized for heterologous expression by using codons more commonly found in *E. coli* (Cosmidis et al., 2015). Synthetic genes were synthesized in one step by PCR from long synthetic oligonucleotides (Gencust service) and inserted in a pET 22b vector (Novagen) at MscI/Bamh1 sites. *E. coli* strain BL21 5(DE3) (Agilent) was transformed by pET vectors with synthetic phosphatase genes. One hundred milliliters of *E. coli* culture were grown in LB medium with 50 μg/ml ampicillin at 37°C, 180 rpm orbital agitation. Expression of phosphatase genes was induced at 30°C by adding 0.5 mM of isopropyl αD-thiogalactoside (IPTG) when cultures reached an OD of 0.8 at 600 nm. After 4 h of expression, *E. coli* cells were centrifuged at 4,000 g and cellular pellets were stored at −20°C.

### Calcification assays

Calcification assays were conducted to assess the capability of *Rta* cells and extracellular extracts to induce the precipitation of Ca-phosphate minerals by enzymatic hydrolysis of a phosphomonoester (glycerophosphate). For that purpose, 30 ml of a 2-week old culture were harvested by centrifugation for 10 min at 4,000 g. The supernatant (extracellular extract) was concentrated 200 times on a 5,000 daltons polyethersulfone membrane inserted in a Millipore Amicon stirred cell and tested for calcification after a hundred-fold dilution. Cell pellets were washed with a 20 mM HEPES buffer at pH 7.5 and concentrated 6-fold. Cells or extracellular extracts were added to a solution composed of 10 mM calcium glycerophosphate and 20 mM HEPES at pH 7.5 (CaGP). No other source of phosphorus (either organic or inorganic) was added. Control assays, without Ca, used 10 mM sodium glycerophosphate and 20 mM HEPES pH 7.5 (NaGP). In order to test the impact of Ca on phosphatase activity with no interference from Ca-phosphate precipitation, additional assays in a medium composed of 10 mM of sodium glycerophosphate as the sole source of phosphorus, 0.75 mM of CaCl_2_ and 20 mM of HEPES at pH 7.5 (NaGP+Ca), were performed. The assays, in either CaGP, NaGP, or NaGP+Ca media, were run for 35 days at 30°C in the dark with shaking.

To test calcification by transformed *E. coli* strains, cells were grown overnight, and harvested by centrifugation for 10 min at 4,000 g. Cell pellets were washed with 20 mM of HEPES buffer at pH 7.5 and resuspended in assay solutions at a concentration of 10^8^ cells/ml. Assays were run for 7 days at 37°C with shaking.

Concentrations of dissolved Ca and inorganic phosphate (orthophosphate) were measured at different times in the assay solutions. For this purpose, half a milliliter of the solutions was sampled at different time intervals. Samples were centrifuged at 6,000 g for 10 min. Supernatants were filtered at 0.2 μm. The obtained dissolved fraction (solute and particles smaller than 0.2 μm) was acidified with 1% HNO_3_ and stored at 4°C. Dissolved calcium concentrations were determined using the cresolphthalein complexone spectrophotometry procedure (Moorehead and Biggs, 1974). Dissolved orthophosphate concentrations were determined using the ascorbic acid spectrophotometry procedure (Chen et al., 1956).

### Phosphatase activity assays

Phosphatase activity was measured using a conventional spectrophotometric assay based on the ability of phosphatases to hydrolyse a derivative of *p*NPP (*p*-nitrophenylphosphate) to *p*-nitrophenol, a chromogenic product with a maximal absorbance at 405 nm (Engvall, 1980). Conversion between absorption at 405 nm and the concentration of released *p*-nitrophenol (hence orthophosphate) was achieved using an extinction coefficient of 18,000 M^−1^ cm^−1^ at 405 nm (Zhang and VanEtten, 1991). Three different *p*NPP derivatives were used as substrates at a concentration of 600 μM: *p*-nitrophenylphosphate monoester (*p*NPP), bis *p*-nitrophenylphosphate diester (bis-*p*NPP) and Thymidine 5′-monophosphate *p*-nitrophenylester (T*p*NPP) as a deoxynucleotide analog.

Assays were performed on total soluble proteins extracted from the bacterial cells in order to ensure an optimal access of the enzymes to the substrates. Total soluble proteins were extracted from stationary cultures of *Rta* (~2 × 10^9^ cells) and *E. coli* (~4 × 10^9^ cells), using the “Bug buster protein extraction” reagent (Novagen), following the protocol recommended by the supplier. This reagent allowed gentle disruption of cell walls, resulting in the release of soluble proteins (including periplasmic proteins). The lysate was cleared by centrifugation at 13,000 g to get the soluble fraction. The phosphatase activities of the total soluble protein of *Rta* and *E. coli* cultures were measured in a buffer containing 50 mM Tris at pH 8, 5 mM of CaCl_2_, 5 mM of MgCl_2_, and 0.6 mM of substrate (*p*NPP, bis-*p*NPP, or T*p*NPP).

The following controls were used. (i) We checked that no spontaneous hydrolysis of the 3 *p*NPP derivatives occurred in the absence of phosphatases under the same chemical conditions as those used for phosphatase activity assays, i.e., after addition of the derivatives to the “Bug Buster protein extraction” reagent in 50 mM Tris, with 5 mM of CaCl_2_, 5 mM of MgCl_2_ and at pH 8.5. (ii) An *E. coli* strain BL21 5(DE3) transformed by a pET vector containing a gene not encoding a phosphatase but a small heat shock protein (Uniprot accession Q38806) was incubated in LB with ampicillin. IPTG was added at 30°C for 4 h when the culture reached an OD of 0.8 at 600 nm. The lysate had no significant phosphatase activity (data not shown). (iii) Last, we used an *E. coli* strain BL21 5(DE3) transformed by a pET vector containing the *E. coli phoA* gene as a positive control. PhoA is a well-known monoester phosphatase (Cosmidis et al., 2015). This strain was processed similarly to the other transformants. We tested the phosphatase activity of the culture lysate after IPTG induction, toward the 3 *p*NPP derivatives.

### Electrophoresis

Protein extracts were loaded on 10% polyacrylamide gels for a denaturing SDS PAGE electrophoresis. Gels and samples were prepared as described by Laemmli (1970). Molecular weight standards and gel staining solution (EZ blue) were purchased from Biorad and Sigma-Aldrich, respectively.

### Calculation of supersaturation indices with respect to hydroxyapatite

The saturation index is defined as the decadic logarithm of the ratio of the ion activity product (IAP) over the solubility constant (Ks): SI = log (IAP/Ks). Dissolved calcium and orthophosphate concentrations were used to calculate the saturation index of the solutions with respect to hydroxyapatite, assuming a pH of 7.5, using the CHESS code (van der Lee, 1998) and a solubility constant for hydroxyapatite K_HA_ as log(K_HA_) = −57.74 (Fujita et al., 2007). Cosmidis et al. (2015) measured that pH remained constant at 7.5 in a calcification assay similar as the one used here. Saturation indices were not calculated when orthophosphate or calcium concentrations were below the detection limits.

### Fourier-transform infrared spectroscopy

Precipitates formed by Rta cells or recombinant *E. coli* cells expressing either *E. coli* PhoA or *Rta* PhoX1 enzymes in CaGP medium were harvested by centrifugation after 35 days (for *Rta*) or 7 days of incubation (for recombinant *E. coli*). Samples were washed in a 20 mM HEPES buffer at pH 7.5 first, then in MilliQ water before drying for 48 h at ~45°C. About 3 mg of sample were gently ground in an agate mortar and mixed with ~300 mg of dried potassium bromide. Samples were pelleted under 9 tons of pressure for ~1 min and dried at ~90°C. After a second pressing, Fourier-Transform InfraRed (FT-IR) spectra were recorded between 400 and 4,000 cm^−1^ with a resolution of 1 cm^−1^ using a Nicolet 7600 FT-IR spectrometer. One hundred spectra were averaged for each sample. The baseline subtraction was done with the program Omnic 7.3. FT-IR spectra of *E. coli* cells grown in LB medium, and a reference hydroxyapatite (Sigma-Aldrich) were measured for comparison. All spectra were normalized to the maximum peak intensity.

### Scanning electron microscopy

Scanning electron microscopy (SEM) analyses were performed using a Zeiss Ultra 55 field emission gun SEM. Images were acquired using an Everhart-Thornley detector at an accelerating voltage of 10 kV and a working distance of ~7 mm or an in column detector (InLens) at 2 kV and a working distance of 1.5 mm. Samples were prepared following the same protocol as Cosmidis et al. (2015). Briefly, cell suspensions were washed and filtered through a 0.2 μm polycarbonate filter (GTTP, Millipore). Filters were then air-dried and coated with carbon, prior to SEM analysis.

### Functional annotation of phosphatase genes

Eight families of non-specific phosphatases were previously described in the literature, with an optimal activity in an alkaline (PhoA, PhoD, PhoK, PhoX) or acid (AcpA, PhoN, AphA, NSAPc) pH range. In order to identify their homologs in the genome of *Rta*, we first selected a set of reference sequences corresponding to bacterial proteins with available 3D structures, one per family (Table 1). In addition, we described each family by a specific set of pre-calculated sequence profiles (PSSMs, for Position-Specific Scoring Matrices). These PSSMs were retrieved from the NCBI Conserved Domain Database (version 3.14) (Marchler-Bauer et al., 2015) according to their similarity to the family reference sequence, using the RPS-Blast program, a standalone version of CD-search (Marchler-Bauer and Bryant, 2004). For each family of phosphatases, we removed the profile redundancy as follows: among the PSSMs whose significant hits on the given reference sequence overlap with a mutual coverage exceeding 70% of the length of the longest hit, only one profile (the one with the lowest *E*-value) was added to the dataset characterizing the family. The final sets of reference PSSMs are listed in Table 1.

**Table 1:** Reference protein dataset of the phosphatase families.

**Phosphatase families**	**Sequence origin**	**Uniprot sequences (accession and length)**	**CDD profiles (accession and hit position)**	**3D structures (PDB code and reference)**
PhoA	*Escherichia coli* K12	P00634 (471)	PRK10518 [1–471]	1alkA Kim and Wyckoff, 1991
PhoK	*Sphingomonas* sp. BSAR-1	A1YYW7 (559)	pfam01663 [44–494]; COG1524 [41–114]	3q3qA Bihani et al., 2011
AcpA	*Francisella novicida* U112	A0Q436 (514)	pfam04185 [55–471]; TIGR03397 [259–464]	2d1gA Felts et al., 2006b
PhoD	*Bacillus subtilis 168*	P42251 (583)	COG3540 [22–539]; cd07389 [174–468]	2yeqA Rodriguez et al., 2014
PhoX	*Pseudomonas fluorescens* Pf0-1	Q3K5N8 (633)	COG3211 [17–633]	4alfA Yong et al., 2014
PhoN	*Salmonella typhimurium*	Q8KRU6 (250)	cd03397 [8–233]; smart00014 [109–217]	2a96A Makde et al., 2007
AphA	*Escherichia coli* K12	P0AE22 (237)	PRK11009 [1–237]	2b82A Calderone et al., 2006
NSAPc	*Bacillus anthracis*	Q81L82 (275)	TIGR01533 [42–272]	2i34A Felts et al., 2006a

*The sequence lengths (in amino acids) are given between parentheses. For each profile accession (CDD, version 3.14), the boundary positions of their hit detected by RPS-Blast on the corresponding reference sequence are indicated between brackets. For each phosphatase family, the profile accessions are ordered according to the E-value (increasing order) of their hits on the reference sequences*.

All translated coding sequences (CDS) of *Rta*, i.e., the putative proteome as provided by the automatic structural annotation of the *Rta* genome (De Luca et al., 2011), were then compared by RPS-Blast with all selected sets of reference PSSMs. In this functional annotation procedure, a given CDS was annotated as a member of a given phosphatase family when it shared at least one significant hit of each PSSM of the family reference dataset. *E*-value threshold was set at 1e-10. In order to remove false positive annotations caused by remote homology, i.e., sequences belonging to the same protein superfamily but laying outside any family of interest, validation was further done by blastp (low stringency search, with 1e-3 as *E*-value threshold) with phosphatase reference sequences as a query.

The entire procedure of functional annotation was also applied to the NCBI set of bacterial genomes completed before March, 2016.

### Prediction of transcription units and detection of regulatory genomic sequences

The *Rta* transcription units encompassing the genes identified as phosphatases using our functional annotation procedure were defined according to DOOR (Mao et al., 2009), a database of computationally predicted operons covering 2072 bacteria genomes. The operon prediction program used to generate this database has been described by Dam et al. (2007) and was ranked to be the best among 14 methods by Brouwer et al. (2008). The prediction accuracy was estimated to be higher than 80% for genomes where only few operons were already known.

Putative transcription factor (TF) binding sites were searched for in the non-coding genomic regions located upstream of the *Rta* phosphatases CDS, or upstream of the first gene of the operons to which they were predicted to belong. For that purpose, we detected significant similarities between these non-coding genomic sequences and sequence profiles describing the binding specificity of known TFs of *E. coli* K-12 (86 PSSMs provided by RegulonDB, Huerta et al., 1998), using the program *matrix-scan* available on the RSAT web server (Turatsinze et al., 2008). Owing to the AT-richness of the prokaryotic regulatory regions whatever the GC content of the genomes (Cordero and Hogeweg, 2009), the large and experimentally validated dataset provided by RegulonDB constituted a reference for the identification of TF binding sites in bacteria. Pairwise hit *p*-values were estimated according to an organism-specific Markov model of order 1, precalibrated on all the non-coding upstream-gene sequences of *Rta* and representing their global oligonucleotide composition (i.e., the background model). The *p*-value threshold was set at 1e-4 (i.e., 1 false positive prediction expected per 10 kb).

### Prediction of protein subcellular location and export pathway

The subcellular locations of *Rta* phosphatases were predicted from their amino acid sequences using the program PSORTb v3.0 (Yu et al., 2010) with Gram-negative bacteria models. In addition, the presence of N-terminal signal peptides, the position of their cleavage site, and the corresponding export pathway were investigated by PRED-TAT (Bagos et al., 2010). Theoretical isoelectric point and molecular weight of the predicted precursor and mature protein forms were computed from their respective sequence by ProtParam tool from ExPASy (http://web.expasy.org/protparam).

### Molecular homology modeling and analysis of protein surface properties

The protein structures of *Rta* phosphatases were modeled using the HHpred server (version 5) (Meier and Söding, 2015). The HMM-HMM similarity search was done against the PDB70 database, as available on the 3rd of January 2015. Models based on optimal multiple templates were automatically built using MODELLER (Šali and Blundell, 1993). The quality of the models was checked by Verify3D (Lüthy et al., 1992). Structures and models were superimposed with Matras (Kawabata, 2003). The 3D coordinate files of the protein models are provided in Supplementary Data Sheet 1.

Electrostatic potential maps of 3D structures and models were computed and drawn using the APBS plugin (Baker et al., 2001) in the PyMOL Molecular Graphics System (version 1.8 Schrödinger, LLC), excluding the charges of the active-site metal ions.

The amino acids involved in noncovalent interactions with P ligand and/or metal ions in the reference 3D structures, according to the CCP4 program *contact* (Winn et al., 2011), were considered as active site residues. After removing ligand, co-factors and putative signal peptide from the 3D coordinate files, POPS server (Cavallo et al., 2003) was used to compute the solvent-accessible surface area (further described as accessibility) of each amino acid. Their sum over the active site residues (or amino acids at homologous positions in protein models) was considered as the active site accessibility.

## Results

### *Rta* phosphatase activities and biomineralization

**Figure 1:**
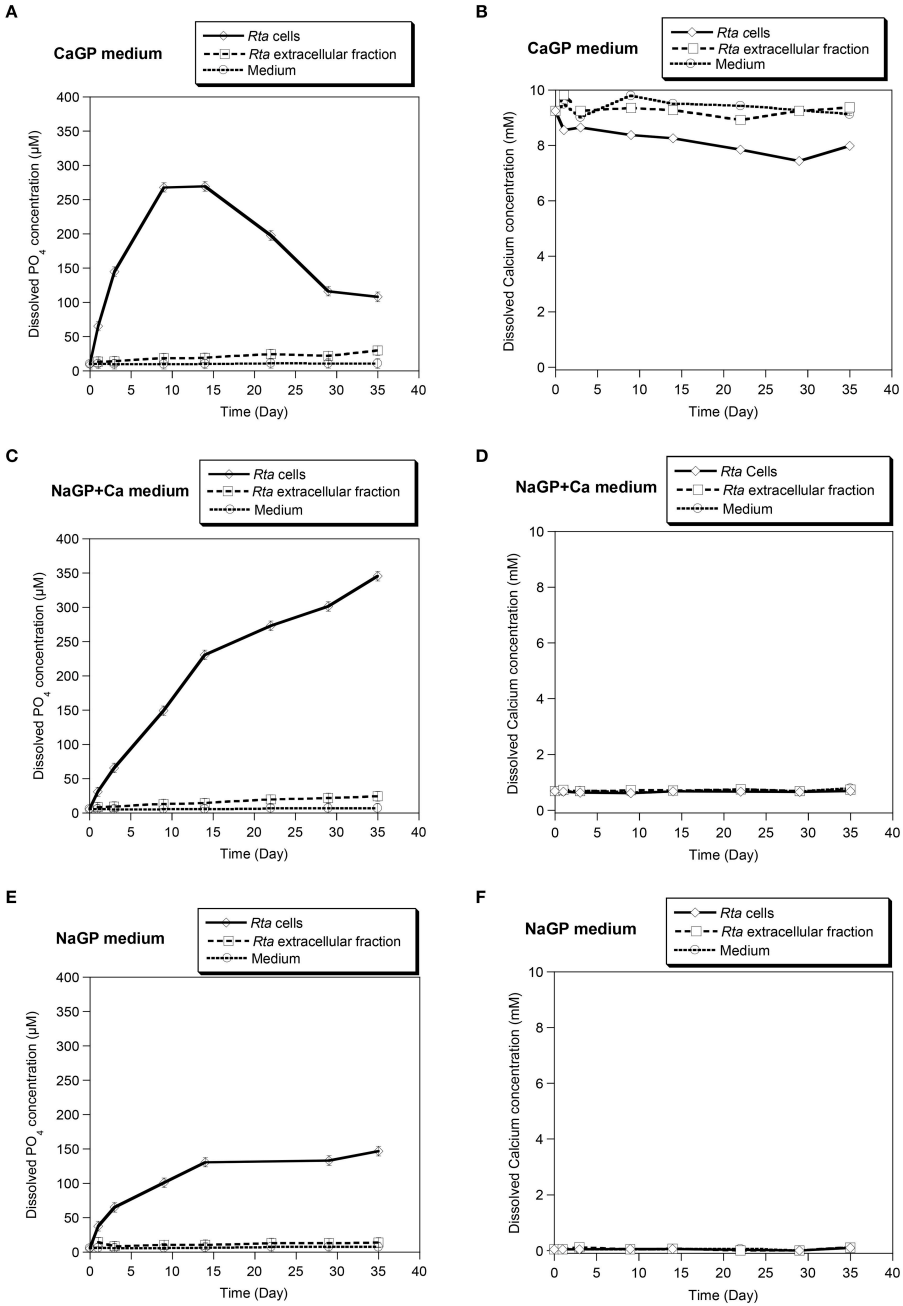
Calcification assay performed in different media on *Rta* cells and the extracellular fraction. Three media were used: **(A,B)** CaGP (10 mM calcium glycerophosphate and 20 mM HEPES at pH 7.5); **(C,D)** NaGP+Ca (10 mM of sodium glycerophosphate, 0.75 mM of CaCl_2_ and 20 mM of HEPES at pH 7.5), and **(E,F)** NaGP (10 mM sodium glycerophosphate and 20 mM HEPES pH 7.5). The NaGP medium was used as a control assay without Ca. For each assay, time variations of the concentrations of dissolved orthophosphates **(A,C,E)** and dissolved Ca^2+^ **(B,D,F)** were measured. Abiotic controls (labeled Medium) consisted in the media without cells. Error bars (smaller than the symbol size) represent the instrumental error: ±0.85 μM and ±50 μM for dissolved orthophosphates and dissolved calcium, respectively.

*Rta* cells were suspended in three different media (CaGP, NaGP, or NaGP+Ca) at a pH of 7.5 with glycerophosphate (GP) as a sole source of phosphorus. Concentrations of dissolved inorganic orthophosphate ions (Pi) and dissolved Ca^2+^ (Ca) were monitored during the course of the experiment (Figure 1).

**Figure 2:**
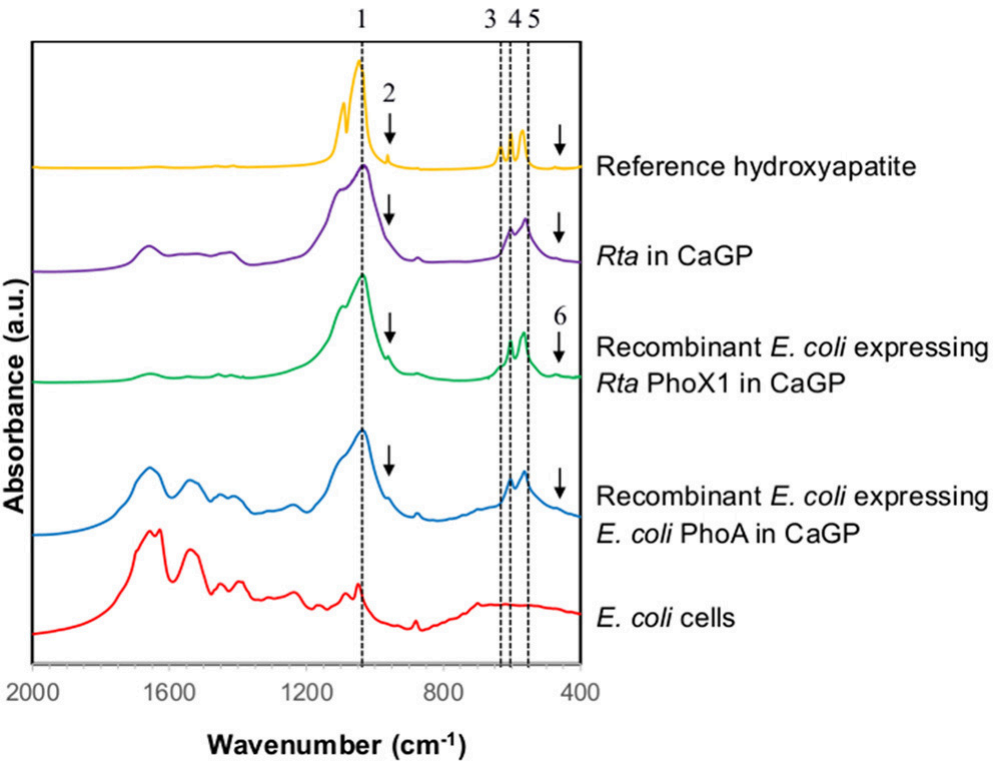
FT-IR spectra of *Rta* and recombinant *E. coli* cells in CaGP medium. The FT-IR spectra of *E. coli* cells (grown in LB medium) and of a reference hydroxyapatite are shown for comparison. The peaks assigned to hydroxyapatite are numbered as follows. 1: 1,035–1,045 cm^−1^ corresponding to P-O stretching ν_3_; 2: 960–962 cm^−1^ corresponding to P-O stretching ν_1_; 3: 630–633 cm^−1^ corresponding to librational mode of OH^−^ groups; 4 and 5: 602 and 563 cm^−1^, respectively, corresponding to P-O-P bending ν_4_; 6: 472 cm^−1^ corresponding to P-OP bending ν_2_.

*Rta* cells as well as to a lower extent, *Rta* extracellular fraction, were able to hydrolyse glycerophosphate (GP), as shown by the increase of Pi concentration over time in all inoculated experiments (Figures 1A,C,E). The increase of Pi concentration was higher for *Rta* cells and *Rta* extracellular fraction incubated in the presence of calcium (CaGP and NaGP+Ca, Figures 1A,C) than without Ca (NaGP, Figure 1E), at least for the 9 first days. Moreover, after 9 days, (i) the concentration of dissolved Pi was significantly higher (267 vs. 149 μM) when cells were incubated with 10 mM Ca (CaGP, Figure 1A) than 0.75 mM Ca (NaGP+Ca, Figure 1C); (ii) for *Rta* cells in CaGP (Figure 1A), Pi concentration plateaued before a decrease down to 116 μM (day 29) due to the precipitation of calcium phosphate. Precipitation in this assay was supported by the net decrease of Ca concentration from 10 mM (CaGP medium, day 0) to 7.4 mM (day 29) (Figure 1B). In contrast, no significant variation of Ca concentration was observed when cells were incubated with 0.75 mM Ca (NaGP+Ca, Figure 1D) or without Ca (NaGP, Figure 1F). Precipitation of Ca phosphates by *Rta* cells in CaGP was also detected by Fourier-Transform InfraRed (FT-IR) spectroscopy analyses (Figure 2). Precipitates were identified as poorly crystalline hydroxyapatite based on the detection of several characteristics vibrational bands at 472 cm^−1^ (P-O-P bending ν2), 563 and 602 cm^−1^ (P-O-P bending ν4), 960–962 cm^−1^ (P-O stretching ν1), and 1,035–1,045 cm^−1^ (P-O stretching ν3).

In contrast, the concentration of dissolved calcium remained constant for cells and extracellular fractions in NaGP+Ca (Figure 1D) as well as extracellular fractions in CaGP (Figure 1B). Based on calculations of the saturation index (SI) of the solutions (Supplementary Table 1), a minimum SI value of 10 was required for precipitation to occur, a condition that was met with *Rta* cells in CaGP only.

**Figure 3:**
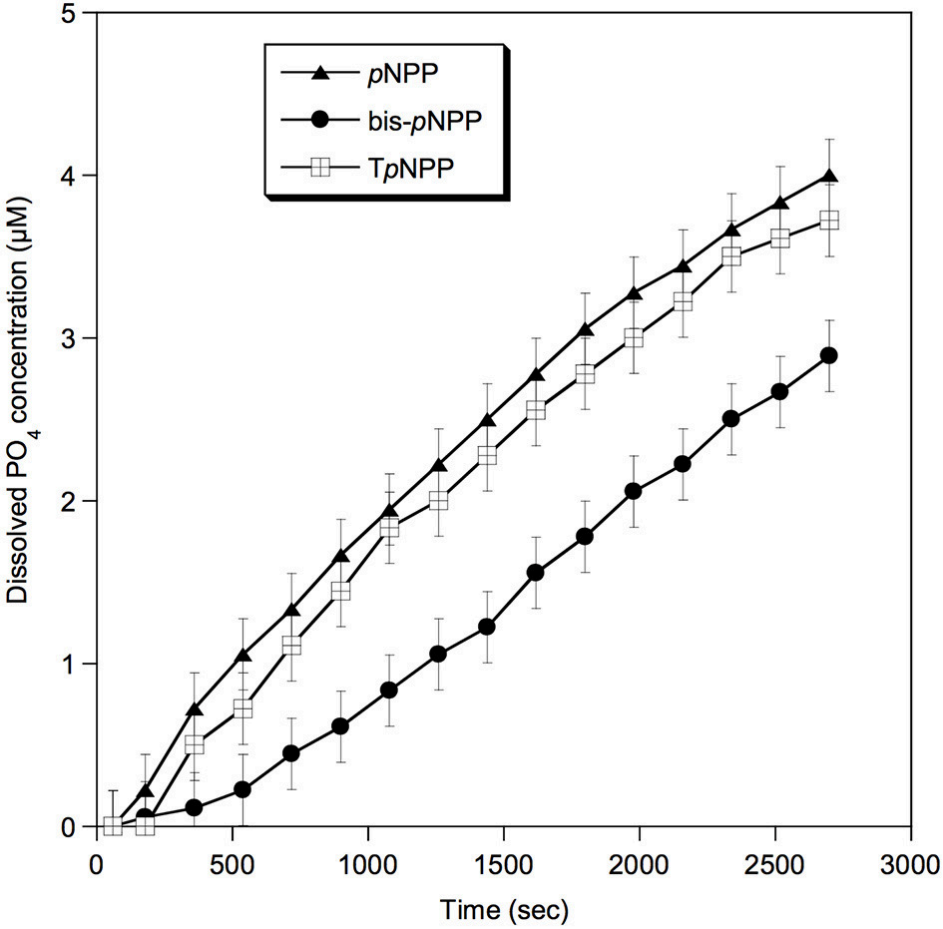
Phosphatase activity of *Rta* cells toward different substrates. *p*NPP, *p*-nitrophenylphosphate (phosphomonoester); bis-*p*NPP, bis *p*-nitrophenylphosphate (phosphodiester); T*p*NPP, Thymidine 5′-monophosphate p-nitrophenylester (phosphodiester, deoxynucleotide analog). Error bars (±0.22 μM) represent the systematic instrumental error.

Furthermore, the phosphatase activity of *Rta* soluble proteins was tested by colorimetric assays in the presence of other organic P substrates: monoester (*p*NPP), diester (bis-*p*NPP), and a diester deoxynucleotide analog (T*p*NPP). A broad phosphatase activity toward the different substrates was detected (Figure 3), the highest rate of reaction being toward monoester and nucleotide analog, corresponding to 1.7 and 1.6 pmol s^−1^ of orthophosphate released, respectively (1.1 pmol s^−1^ toward bis-*p*NPP).

### *In Silico* prediction of *Rta* non-specific phosphatases

We identified 5 putative phosphatases in the genome of *Rta*, according to their sequence similarity with reference profiles and sequences of known phosphatase families (Supplementary Table 2). They belonged to 2 non-homologous families of alkaline phosphatases: 1 PhoD and 4 PhoX, further described as PhoX1-4. No member of other known alkaline phosphatase families, i.e., PhoA and PhoK, and no acid phosphatase homolog were detected. The *phoD* gene product was already described as a candidate alkaline phosphatase D precursor in the latest annotation release available for the *Rta* genome from the NCBI. In contrast, no relevant functional annotation was available for the 4 putative PhoX family members, described as (conserved) hypothetical proteins.

The sequences of the 5 putative *Rta* phosphatases exhibited the same protein domain composition than the reference of their respective families (Supplementary Table 2). No additional phosphatase gene (or pseudogene) was found in the noncoding part of the *Rta* genome by similarity search of the reference sequences using tblastn.

### Genomic organization, regulatory sequences, and predicted cellular location of *Rta* phosphatases

The *phoD* gene was located on the direct DNA strand, while the 4 *phoX* genes were located on the reverse strand (Table 2). These 5 genes were distant from each other on the genome, i.e., there was no joint organization as gene clusters or operons. The *phoX2* gene was predicted as part (3′-end) of an operon in the DOOR database (Mao et al., 2009), together with two other genes: from 5′ (locus tag Rta_37630) to 3′ (Rta_37620), the protein sequences corresponding to these genes were described as (1) uncharacterized (Uniprot accession F5Y2Z8; similar to non-heme di-iron oxygen transport proteins, CDD profile pfam01814) and (2) cytochromes P450-like (Uniprot accession F5Y2Z7), respectively. The 4 other phosphatase-encoding genes were considered as complete transcription units.

**Table 2:** Genomic sequence features of the *Rta* phosphatase genes and their neighboring regions.

	**Genes (CDS)**				**Length of Upstream (U) and Downstream (D) regions**		**Prediction of cis-regulatory elements: RSAT** ***matrix-scan*** **hits**		
**Protein name**	**Locus tag**	**DNA strand**	**Start**	**End**	**U (nt)**	**D (nt)**	**TF name**	**Upstream sequence positions**	***p*-value**
PhoD	Rta_17200	D	1805367	1806959	148	19	–	–	–
PhoX1	Rta_14460	R	1514607	1512676	109	21	–	–	–
PhoX2	Rta_37610	R	4006412	4004532	74*	200	–	–	–
PhoX3	Rta_37000	R	3936191	3933921	114	188	PhoB	[−96, −81]	5.3e-05
							Fur	[−18, −1]	2.3e-05
PhoX4	Rta_01350	R	134200	132824	416	48	FadR	[−173, −158]	1.7e-05
							Cra	[−149, −132]	5.7e-05
							FhlA	[−125, −112]	6.9e-05

*Genes are located on direct (D) or reverse (R) DNA strands; the CDS start and end refer to base positions on the direct strand. Operon-included region is indicated by an asterisk (according to the predicted list of operons available in the DOOR database (Mao et al., 2009). Transcription factor (TF) names are those used in RegulonDB (Huerta et al., 1998); Fur, Ferric uptake regulation; PhoB, phosphorus uptake and metabolism regulation; Cra, Catabolite repressor regulator; FhlA, Formate hydrogen lyase activator; FadR, Fatty acid degradation and metabolism regulator*.

In order to predict the regulatory pathways controlling the expression of phosphatases in *Rta*, and assess whether they were expressed constitutively or under controlled regulation and in the latest case which environmental parameters may impact their transcriptional regulation, we looked for putative regulatory elements. We detected putative cis-regulatory elements that may control the transcription of the *phoX3* and *phoX4* genes, based on their similarity with known transcription factor (TF) binding sites in *E. coli* K-12. The significant hits (Table 2) corresponded to 5 TF, the complete (PhoB, Fur, FadR) or partial (Cra, FhlA) homologs of which were identified in the *Rta* genome by blastp (Supplementary Table 3). No cis-regulatory element was identified in the upstream region of the other putative phosphatase genes, or in the upstream region of the first gene of the *phoX2* operon.

While belonging to the same family, the 4 putative PhoX proteins strongly varied in their sequence length, ranging from 458 a.a. (PhoX4) to 756 a.a. (PhoX3) (Table 3). The 5 *Rta* phosphatases were predicted by PRED-TAT to share a N-terminal signal peptide (length ranging from 31 to 67 a.a.) with specific motifs recognized by the twin-arginine translocase (tat). This bacterial system exports folded proteins to non-cytoplasmic cell space. In parallel, different cell locations were predicted by PSORT-B: extracellular (PhoD), non-cytoplasmic (PhoX2-4) and cytoplasmic (PhoX1). Therefore, there was a conflict between PSORT-B and PRED-TAT predictions for PhoX1, since cytoplasmic proteins do not contain signal peptides.

**Table 3:** Protein sequence features of the *Rta* phosphatases and predicted subcellular location.

**Protein name**	**Sequence accession (Uniprot)**	**Length (aa)**	**Predicted location (PSORTb)**	**Predicted signal peptide: length, export pathway (PRED-TAT)**	**MW (kDa)**	**Theoretical pI**
PhoD	F5XVP7	530	Extracellular	L = 31, Tat signal	58.8 (55.5)	7.3 (6.9)
PhoX1	F5Y472	643	Cytoplasmic	L = 51, Tat signal	69.5 (64.1)	6.3 (6.3)
PhoX2	F5Y2Z6	626	Non-cytoplasmic	L = 50, Tat signal	67.2 (62.3)	9.3 (9.2)
PhoX3	F5Y248	756	Non-cytoplasmic	L = 67, Tat signal	80.4 (73.7)	5.4 (5.6)
PhoX4	F5Y3B1	458	Non-cytoplasmic	L = 31, Tat signal	48.9 (45.7)	5.3 (4.9)

*Molecular weight (MW) and isoelectric point (pI) are indicated for both precursor and mature forms of each protein (the latter being between parentheses), according to the predicted cleavage site*.

The theoretical pI of the mature forms of Pho proteins were acid (PhoX1, PhoX3-4), alkaline (PhoX2) and near-neutral (PhoD) ranging from 4.9 to 9.2. Predicted signal sequences did not significantly affect the theoretical pI of *Rta* phosphatases (Table 3). Finally, the molecular weight of the predicted mature forms ranged from 45.7 (PhoX4) to 73.7 kDa (PhoX3).

### Diversity of the physicochemical properties of *Rta* phosphatases

**Figure 4:**
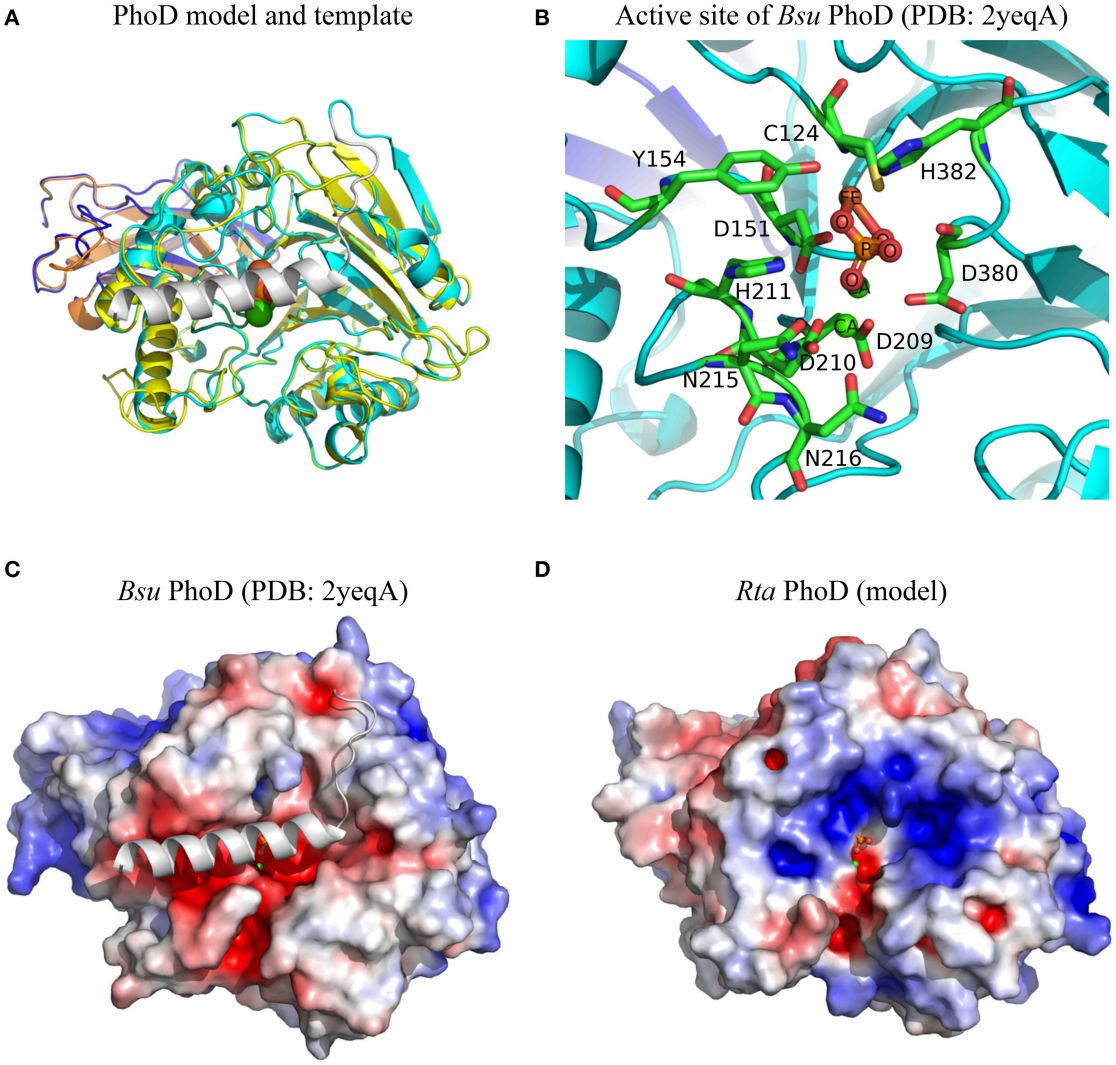
Structural features of *Rta* PhoD model. **(A)** Superimposition of 3D model of *Rta* PhoD and 3D structure of *Bsu* PhoD used as template (PDB chain 2yeqA). *Bsu* PhoD: Ig-like domain and catalytic domain are colored in dark blue and cyan, respectively; C-ter cap in gray. *Rta* PhoD: Ig-like domain (orange), catalytic domain (yellow). **(B)** 3D view of the active site of *Bsu* PhoD. The amino acid residues are labeled according to their PDB numbering. Atom name of co-factors and inorganic phosphate molecule are labeled. Hydrogens are hidden. **(C,D)** Electrostatic potential maps of *Bsu* PhoD without C-ter cap and *Rta* PhoD. The surface potentials are contoured from −5 kT/e (red) to 5kT/e (blue). All structures are shown according to their orientation in the superimposition. The positions of ligand and ions are those from *Bsu* PhoD; their atoms are represented as spheres of given van der Waals radius **(A)** or small fixed radius **(B–D)**.

A molecular model of *Rta* mature PhoD (499 amino acids) was built by homology with the 3D structure of *Bacillus subtilis* (*Bsu*) mature PhoD (527 amino acids), which measured the exact same size as *Rta* PhoD plus the 28 amino acids of a C-ter cap, not found in Rta PhoD. Both *Rta* and *Bsu* mature PhoD shared 45% sequence identity. The root mean square deviation (RMSD) between the C-alpha atoms of the two superposed structures was 0.48 Å (Figure 4A). Based on this model, 9 of the 10 residues of the active site located in the catalytic domain of *Bsu* PhoD (Figure 4B) were conserved in *Rta* PhoD, whose active site was composed as follows (sequence numbering): C154, D181, Y184, D235, D236, H237, N241, D242 (N216 in *Bsu* PhoD, PDB numbering), D425, H427. Two residues of *Bsu* PhoD active site, labeled R506 and H510 and belonging to a flexible C-terminal extension (C-ter cap, 28 amino acid length), had no equivalent in *Rta* PhoD (Supplementary Image 1A).

**Figure 5:**
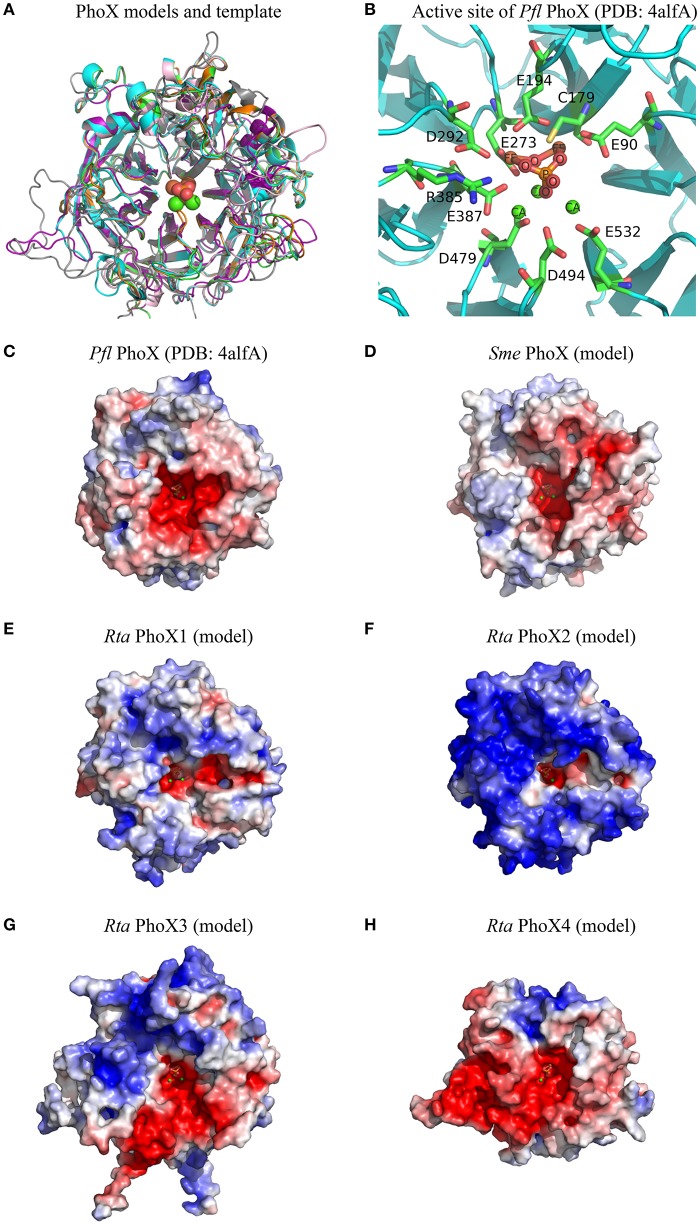
Structural features of *Rta* PhoX1-4 models. **(A)** Superimposition of 3D models of *Sme* PhoX (pink), *Rta* PhoX1 (green), *Rta* PhoX2 (orange), *Rta* PhoX3 (gray), *Rta* PhoX4 (purple) and 3D structure of *Pfl* PhoX used as template (PDB chain 4alfA, cyan). **(B)** 3D view of the active site of *Pfl* PhoX. The amino acid residues are labeled according to their PDB numbering. Atom name of co-factors and inorganic phosphate molecule are labeled. Hydrogens are hidden. **(C–H)** Electrostatic potential maps. The surface potentials are contoured from −5 kT/e (red; −15 kT/e for *Sme* PhoX) to 5kT/e (blue). All structures are shown according to their orientation in the superimposition. The positions of ligand and ions are those from *Pfl* PhoX; their atoms are represented as spheres of given van der Waals radius **(A)** or small fixed radius **(B–H)**.

Molecular models of mature *Rta* PhoX1-4 were built based on the 3D experimental structure of *Pseudomonas fluorescens* (*Pfl*) PhoX (Figure 5A). This 3D template was also used to generate the molecular model of *Sinorhizobium meliloti* (*Sme*) PhoX (Uniprot accession M4MZI0), whose profile of substrate affinity was experimentally determined by Zaheer et al. (2009). The pairwise RMSD and the pairwise sequence identity of *Pfl* PhoX with each of the PhoX models were: 0.48 Å, 49.5% (*Rta* PhoX1); 0.46 Å, 49.9% (*Rta* PhoX2); 0.69 Å, 40.2% (*Rta* PhoX3); 1.37 Å, 25.4% (*Rta* PhoX4); 0.62 Å, 41.5% (*Sme* PhoX). While *Rta* PhoX1 and PhoX2 exhibited similar sequences (65.7% identity) and structures (0.36 Å RMSD), large insertions and deletions occurred in loops in *Rta* PhoX3 and PhoX4, respectively. Based on these models, the 10 residues of the active site of *Pfl* PhoX (Figure 5B) were conserved in the *Rta* PhoX proteins and also in *Sme* PhoX (Supplementary Image 1B).

Based on these 3D structures and models, we computed the solvent-accessible surface area of each amino acid located in the protein active site. For a given protein, the sum of these areas over the active site residues (or amino acids at homologous positions in protein models) is called the active site accessibility. This parameter correlates to the protein surface that can accommodate the substrate during the enzymatic process. The accessibility of the active site of the 4 *Rta* PhoX proteins strongly varied, ranging from small (PhoX2, 213.5 Å^2^) to large values (PhoX1 and PhoX4, 302.0 and 333.9 Å^2^, respectively) with an intermediate value for PhoX3 (270.6 Å^2^) close to the reference *Pfl* PhoX (268.7 Å^2^). By comparison, the accessibility of the active site of *Sme* PhoX was 267.7 Å^2^. The active site of *Rta* PhoD exhibited similar accessibility (273.0 Å^2^) to that of the references *Bsu* PhoD (261.6 Å^2^ in an open state, i.e., without its C-ter cap) and *Pfl* PhoX.

Finally, all 3D structures studied here showed active sites with a negative surface net charge (Figures 4C,D, 5C–H). This likely controls their ability or requirement to interact with metal cations (i.e., co-factors). However, the regions surrounding the active sites exhibited variable patterns of surface electrostatic potential, corresponding to negatively charged patches for *Bsu* PhoD, *Pfl* PhoX, *Sme* PhoX, and *Rta* PhoX4 or a mosaic of patches with apolar or distinct charged properties for *Rta* PhoD and *Rta* PhoX1-X3.

### Recombinant expression of *Rta* phosphatases in *E. coli*; phosphatase and calcification activities

Recombinant expression was achieved for 3 of the 5 *Rta* phosphatase genes: *phoD, phoX1*, and *phoX3*. *E. coli phoA* was also cloned in the same vector and tested for expression as a control. On SDS-PAGE, an intense band was systematically observed for each transformed strain in the “IPTG” condition that was absent from the “no-IPTG” condition (Supplementary Image 2). These proteins had apparent molecular masses close to those predicted by *in silico* analyses for the different phosphatases. For *E. coli phoA*, the overexpression of PhoA was confirmed using Western blot with a commercial anti *E. coli* alkaline phosphatase antibody (data not shown). Two proteins were clearly visible for PhoA after an induction time of 4 h, corresponding to the expected molecular weights of the precursor and mature forms of this protein. In contrast, no such precursor forms were observed on SDS-PAGE for the *Rta* PhoD, PhoX1 and PhoX3.

**Figure 6:**
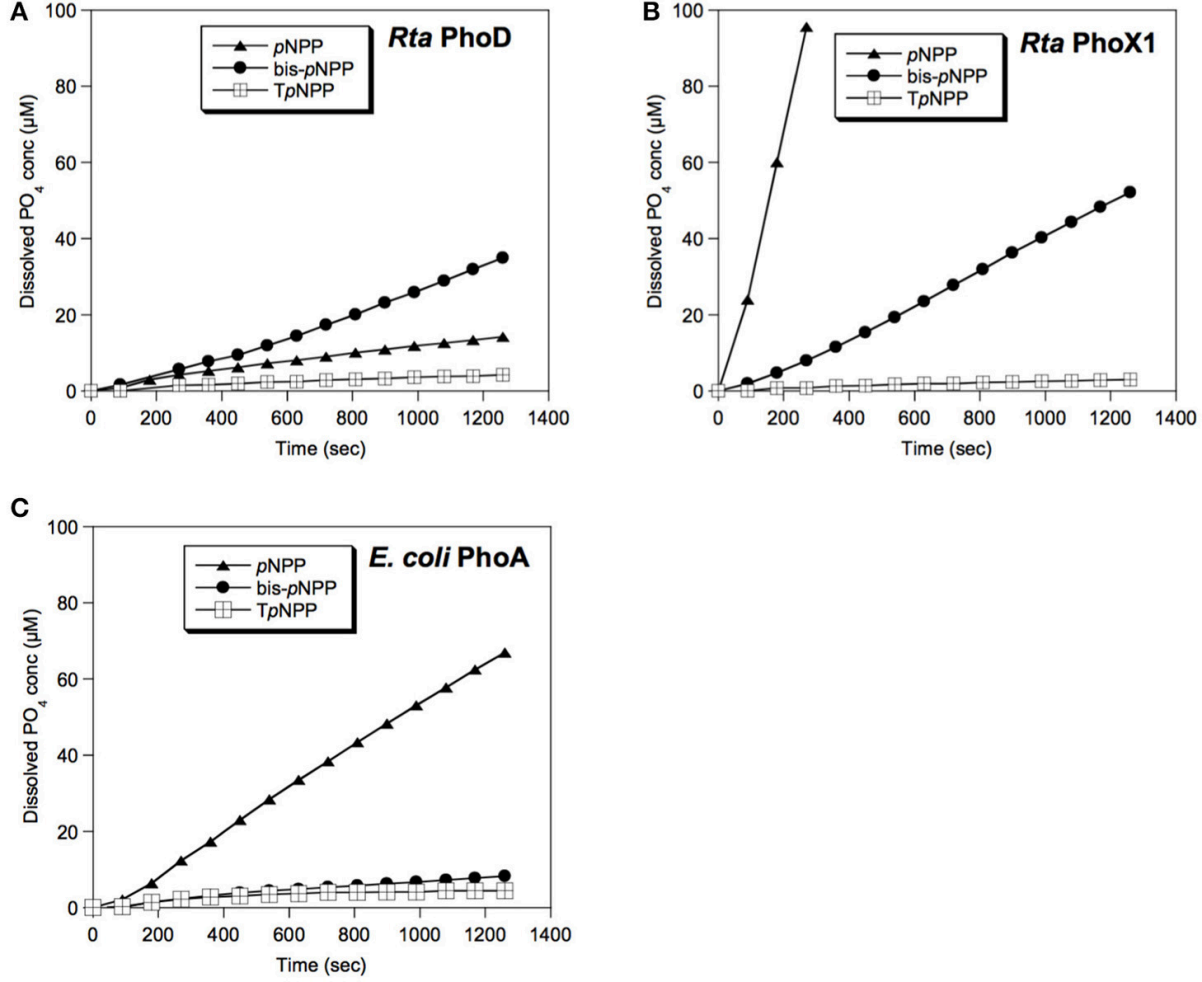
Enzymatic activities of *Rta* phosphatases. The soluble protein fractions extracted from *E. coli* clones overexpressing **(A)** *Rta phoD* or **(B)** *Rta phoX1* were assayed for phosphatase activity toward monoester (*p*NPP), diester (bis-*p*NPP) or nucleotide analog (T*p*NPP) substrates. **(C)** Control assay of the soluble protein fraction of *E. coli* clones over-expressing *E. coli phoA*. Error bars (±0.22 μM, smaller than the symbol size) represent the systematic instrumental error.

The *phoX3* expression was unstable, i.e., the recombinant protein was rapidly degraded over the duration of expression. Recombinant PhoX2 and PhoX4 proteins were toxic in *E. coli* host cells, i.e., they inhibited *E. coli* growth. Therefore, the activities of these 3 proteins could not be measured. Phosphatase assays were carried on total soluble proteins extracted from the *phoD, phoX1* and *phoA E. coli* clones (Figure 6). PhoD and PhoX1 hydrolyzed all the *p*NPP derivatives (*p*NPP, bis-*p*NPP, and T*p*NPP). However, the activity of PhoD was higher for bis-*p*NPP diester (28.2 pmol s^−1^ of orthophosphate released) than *p*NPP monoester (11.1 pmol s^−1^) and T*p*NPP nucleotide analog (3.3 pmol s^−1^), whereas the activity of PhoX1 was higher for monoester (358.6 pmol s^−1^) than diester (43.1 and 2.3 pmol s^−1^ for bis-*p*NPP and T*p*NPP, respectively).

**Figure 7:**
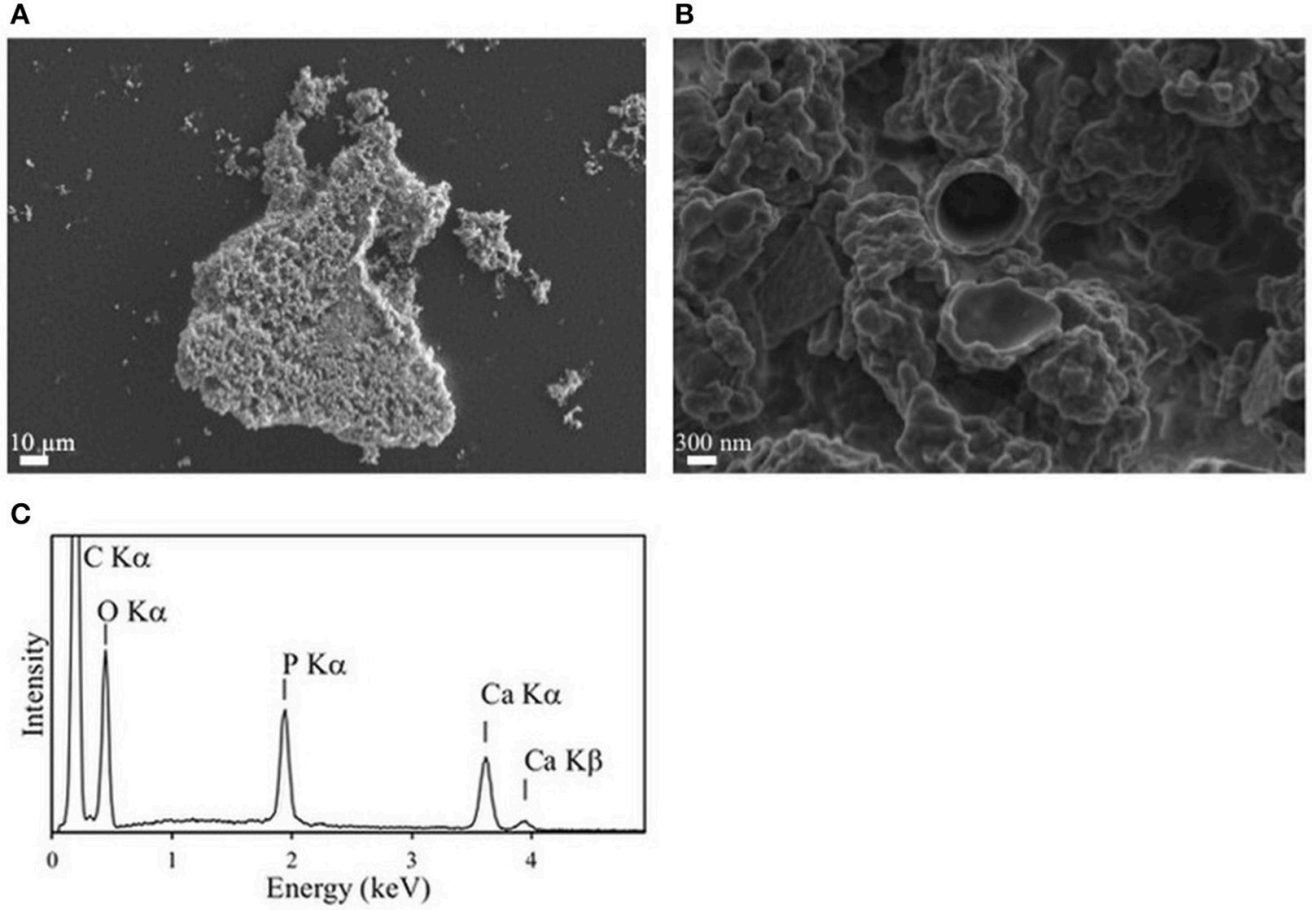
Mineralization of calcium phosphate induced by over-expression of *Rta* PhoX1 in *E. coli* clones. **(A,B)** SEM images in the secondary electron mode. **(A)** Image of a cluster of cells encrusted by calcium phosphate. **(B)** Close-up showing the lumen of a cell in the center. The wall is encrusted by calcium phosphate precipitates. **(C)** Energy dispersive x-ray spectrometry (EDXS) spectrum measured on the mineral phase precipitated on the cells. Emission lines of O, P, and Ca correspond to hydroxyapatite. The emission line of C is due to the cells and the carbon filter on which the sample was deposited.

*E. coli* cells expressing *Rta phoX1* induced hydroxyapatite precipitation in the presence of 10 mM of CaGP as shown by FT-IR spectroscopy (Figure 2), or SEM coupled with energy dispersive x-ray spectrometry (Figure 7). The precipitation of hydroxyapatite induced by *E. coli* cells expressing *E. coli phoA*, previously described by Cosmidis et al. (2015), was confirmed. The main peaks in the FT-IR spectrum of the samples could be unambiguously assigned to hydroxyapatite, with broader bands (e.g., P-O stretching ν_3_ at 1,035–1,045 cm^−1^) than in the reference hydroxyapatite spectrum, suggesting more disordered structures (Cosmidis et al., 2015). Moreover, the P-O stretching ν_1_ (960–962 cm^−1^) was less defined in the “Rta in CaGP” spectrum than in the “Recombinant” spectra, which may indicate a lower crystallinity as well. In contrast, no hydroxyapatite precipitation was evidenced for *E. coli* cells expressing *phoD*.

## Discussion

### Broad substrate recognition by *Rta* phosphatases

*Rta* cells showed a significant phosphatase activity toward a broad spectrum of substrates. While the highest reaction rate was observed for a phosphate monoester and for the nucleotide-analog phosphodiester (T*p*NPP), there was also a significant hydrolysis of bis-*p*NPP, a phosphate phosphodiester molecule smaller than T*p*NPP. Five genes of non-specific phosphatases were identified in *Rta* genome, belonging to the two non-homologous families *phoD* (1 gene) and *phoX* (4 genes, *phoX1-4*). Among the 5 phosphatases, *phoD* and *phoX1* heterologously expressed in *E. coli* were found to catalyze the hydrolysis of different substrates, including diesters at the exception of the nucleotide analog.

In agreement with our observations, PhoD family members have previously been shown to hydrolyze phosphomonoesters and phosphodiesters with a preference for the latter (Rodriguez et al., 2014).

PhoX1 showed a high phosphomonoesterase activity. Therefore, *Rta* PhoX1 profile for organic P substrate (mono- and diesters) was intermediate between the monoester-specific activity of *P. fluorescens* PhoX (Yong et al., 2014), and the affinity of *S. meliloti* PhoX for most of the metabolites with phosphoester bonds, including nucleotides, carbohydrates and amino acids (Zaheer et al., 2009). The 3D model of the *S. meliloti* PhoX (Figure 5D) revealed (i) an active site accessibility similar to *P. fluorescens* PhoX but smaller than *Rta* PhoX1, and (ii) a surface with a high negative net charge, while *P. fluorescens* PhoX and *Rta* PhoX1 both exhibited a surface with a low positive net charge.

While sharing a conserved active site, PhoX proteins showed different patterns of predicted surface charges and accessibility of the active site. The substrates used in the present study corresponded to diverse shapes and occupancies, and were negatively charged: −4 for T*p*NPP, −3 for bis-*p*NPP, and −2 for *p*NPP and GP. It has been shown that positive charges at the surface of the phosphatases may facilitate the loading of negatively charged substrates into their catalytic site (Coleman, 1992). The diversity of the substrate profiles observed within the PhoX family thus seems to rely more on the physicochemical properties of the amino acids surrounding the active site and little on the active site residues themselves.

The capability of *Rta* to hydrolyze nucleotide-analog phosphodiesters was therefore likely due to one of the 3 other phosphatases identified in the genome. The ability of recombinant PhoX2 and PhoX4 to hydrolyze the nucleotide phosphodiester bonds can be discussed, although it could not be verified experimentally. These proteins were encoded by genes with a complete structure and without genomic features related to pseudogenes. According to 3D models, PhoX4 had an active site with a very large solvent accessibility surface (~330 Å^2^) and therefore appeared as a good candidate for the accommodation of large substrates such as nucleotides. The predicted high positive net charges at the surface of PhoX2 (Figure 5F), together with the predicted Tat signal, which should induce the expression of a folded protein in the cytoplasm (Teter and Klionsky, 1999), may explain the toxicity of this protein, which may catalyze DNA degradation when overexpressed in *E. coli*. *PhoX2* was organized into a 3-genes transcription unit (operon). This genomic organization suggests that the proteins encoded by these genes may form a macromolecular assembly when expressed altogether in *Rta* (Wells et al., 2016). In this macromolecular assembly, the catalytic site of PhoX2 may be buried, precluding DNA damage within *Rta* cells. In the present study, *Rta phoX2* only was cloned in *E. coli* without the two other genes of the predicted operon. Moreover, no homolog of these two genes were found in the genome of *E. coli*. As a result, the catalytic site of PhoX2 would not be buried in a macromolecular assembly in *E. coli* and would therefore be toxic to the cells. However, we did not find any report in the literature about such an assembly between homologs of these three proteins and therefore further work will be needed to assess the above assumption.

Overall, the diversity of phosphatases in *Rta* allows the hydrolysis of a broad range of organic P substrates and therefore the release of Pi under diverse trophic conditions. Since Pi release is key to the achievement of high saturation levels with hydroxyapatite and the induction of phosphatogenesis, *Rta* appears as a particularly efficient driver of this process as shown experimentally. Hydroxyapatite precipitation was shown to first occur in the periplasm of *Rta* cells, then in the cytoplasm (Benzerara et al., 2004). The prediction of a non-cytoplasmic location for most of the phosphatases is consistent with these observations, except PhoX1 for which location remains unclear.

### *Rta* harbors an unusual set of diverse phosphatases

The diversity of alkaline phosphatases in microorganisms was previously investigated at the community level by metagenomics, with a particular focus on the relationship between gene abundance and the availability of inorganic phosphorus in ocean surface waters (Luo et al., 2009; Martiny et al., 2009; Sebastian and Ammerman, 2009; Temperton et al., 2011) or in soils (Lidbury et al., 2017; Neal et al., 2017). Overall, PhoX and PhoD families were evidenced to be more abundant than PhoA in high-pH soils and under marine oligotrophic conditions. Interestingly, (i) the Tataouine sand from which *Rta* was isolated showed similar P-depleted and Ca-rich conditions (Gillet et al., 2000), and (ii) the *Rta* genomic repertoire contained phosphatases of both the PhoX and PhoD families. These data support the existence of a relationship between the availability of Ca and P in the environment and the relative abundance of the phosphatase gene families. However, the taxonomic scope of studies on ocean surface waters was limited by the numerical domination of microbial phototrophs, in particular Cyanobacteria, and heterotrophic Alpha- and Gamma-proteobacteria in these environments.

Here, in order to limit the bias due to a limited taxonomic sampling, we annotated the phosphatase genes in the large amount of available bacterial complete genomes. This dataset comprised 3630 genomes from 33 phyla. Eight families, either alkaline or acid phosphatases, were annotated according to the procedure previously applied to *Rta* genome. Compared to the statistical trends that emerged from the analysis of bacterial genomes, the functional potential of the *Rta* genome for phosphatases exhibited atypical features: (i) among the 12 bacterial genomes with at least 4 PhoX genes, *Rta* was the only member of Proteobacteria, a phylum which was represented by 1794 genomes; (ii) while 62% of the bacterial genomes had at least one gene of alkaline phosphatase (71% of the 261 Betaproteobacteria genomes), only 4% (1% for Betaproteobacteria) had at least 5 genes, similarly as *Rta*; (iii) although it was absent in *Rta*, the PhoA family was the most widely represented with 35% of the bacterial genomes exhibiting at least one gene of this family. It was less than 25% for each other family, either alkaline or acid, e.g., 22 and 17% in the case of PhoX and PhoD, respectively. Among the genomic features of bacteria, the linear correlation between the genome size and the global number of genes (abundance) was well known (Hou and Lin, 2009) (Supplementary Image 3; *R*^2^ = 0.97). Though the abundance of phosphatase genes was related to the genome size in our dataset (Kruskal-Wallis test, *p* < 2.2e-16), *Rta* represented the bacterial genome with the highest density of PhoX genes (Supplementary Image 4). In addition, only 0.9% of the bacterial genomes had a higher density of alkaline phosphatase genes than *Rta*. The variable physicochemical properties of *Rta* phosphatases suggested a functional diversification of the assimilation of organic P substrates, driven by P-depleted conditions.

### Environmental parameters involved in the regulation of the phosphatase activity of *Rta*

Our results highlighted the influence of several chemical parameters on the phosphatase activity of *Rta*.

(1) First, phosphomonoester hydrolysis by *Rta* cells was favored by extracellular Ca^2+^. Accordingly, the requirement of calcium ions for phosphatase activity was previously described for PhoD or PhoX proteins (Zaheer et al., 2009; Rodriguez et al., 2014; Yong et al., 2014). The molecular modeling of the *Rta* phosphatases revealed the high-level of conservation of the active site residues within PhoD and PhoX families. Based on this high-level of conservation of the active site residues, the cofactors of the reference proteins, i.e., Ca^2+^ and Fe^3+^, were predicted as probable cofactors of the 5 *Rta* phosphatases. Although there is no experimental or thermodynamic confirmation of this prediction based on sequence conservation, it is interesting to note that Ca was abundant in the sandy soil from which *Rta* was isolated (Gillet et al., 2000). Calcium can thus have a double role in phosphatogenesis: (i) by directly participating to the precipitation of Ca-phosphates but also (ii) indirectly by affecting the activity of some phosphatases. In the first case, the extracellular Ca matters, while intracellular Ca plays a role in the second case. Calcium is a key regulator in eukaryotes and bacteria that can modify the cell state and the pattern of gene expression (Dominguez, 2004; Domínguez et al., 2011). Therefore, the relationship between Ca availability in the environment and the cell induction of phosphatogenesis may overall be complex and depend on processes regulating Ca homeostasis within cells.

(2) In addition, putative cis-regulatory elements similar to known transcription factor binding sites in *E. coli* K-12 were found in *Rta* genome in the upstream region of the PhoX3 and PhoX4 genes. In *E. coli* K-12, these transcription factors are involved in the regulation of the expression of genes related to (i) P uptake and metabolism (*pho* regulon), controlled by the extracellular concentration of inorganic P (PhoB) (Hsieh and Wanner, 2010; Santos-Beneit, 2015), (ii) ferric iron homeostasis (Fur) (da Silva Neto et al., 2009; Porcheron et al., 2013), and (iii) fatty acid and phospholipid metabolism (FadR) (Iram and Cronan, 2005; Fujita et al., 2007). We detected complete homologs of these transcription factors in *Rta* genome. Previously, the alkaline phosphatases of different families have been experimentally identified as members of the *pho* regulon, in particular in the Gammaproteobacteria *E. coli* (Torriani, 1960; Wanner, 1990), *S. meliloti* (Bardin and Finan, 1998; Yuan et al., 2006) and *P. fluorescens* (Monds et al., 2006), as well as in *B. subtilis* (Eder et al., 1996; Hulett, 1996; Prágai et al., 2004), *Mycobacterium tuberculosis* (Torres et al., 2001) and *Synechocystis* sp. PCC 6803 (Suzuki et al., 2004). However, the predicted relationship between iron or fatty acids/phospholipids and non-specific phosphatases at the transcription level may be a specificity of *Rta*. Such a connection between iron uptake, fatty acids/phospholipid metabolism and phosphatase expression may be due to the fact that these phosphatases need Fe as a co-factor and use phospholipids as substrates. However, the ability of *Rta* phosphatases to use phospholipids as organic P substrates in P-depleted conditions will have to be evaluated. Interestingly, in addition to being predicted as a probable cofactor of the *Rta* phosphatases, Fe was also present in the environment where *Rta* was isolated from, although mobilization under oxic conditions likely requires the presence of organic chelatants (Benzerara et al., 2005, 2006). Moreover, fatty acid metabolism has been shown to increase slightly extracellular pH around cells and impact CaCO_3_ biomineralization (Marvasi et al., 2010). Together with Pi release, the same pH effect may play a synergistic positive role on phosphatogenesis, which will have to be further explored experimentally. Overall, the profile of P substrates hydrolyzed by *Rta* cells may thus be tuned by the expression of a varying combination of phosphatases depending on the environmental parameters.

In conclusion, the diverse set of phosphatases found in *Rta* suggests that this strain can hydrolyze diverse P-containing organic molecules under diverse environmental conditions. Since the resulting production of orthophosphates can induce the precipitation of metal-phosphates under some conditions, *Rta* may therefore be particularly prone to phosphatogenesis. Such a relationship between phosphatase diversity within a bacterial strain and its role in phosphatogenesis has not been investigated before. However, the approach developed here combining *in silico* and *in vitro* analyses sets the foundations for future studies of more complex environmental communities with the aim of inferring their phosphatogenesis capabilities.

## Author contributions

FS-P, KB, and ED designed the experiments, ED and GC performed bioinformatics analyses, KB and JC performed SEM analyses, JC and FS-P acquired and interpreted FT-IR spectra, FS-P and CF performed microbial cultures, heterologous expression, and assays, GD and TH provided *Rta* strain and contributed to microbial cultures. All authors contributed to manuscript writing and revision.

### Conflict of interest statement

The authors declare that the research was conducted in the absence of any commercial or financial relationships that could be construed as a potential conflict of interest.
