# Supplementary material for: In Vitro and in Silico Evidence of Phosphatase Diversity in the Biomineralizing Bacterium Ramlibacter tataouinensis
Source: Front Microbiol. 2018 Jan 11;8:2592. doi: 10.3389/fmicb.2017.02592 (PMC5768637; doi:10.3389/fmicb.2017.02592)
Supplement: Supplementary file 1 [file Image1.pdf]

a

```
[AVE_SECSTR] CCCCSSCsCTTCEEEEECCCSSCEEEEEEECcSBcsttTscccsSCEEEEEEEEESSTTsSSEEEEEEEEEECtttTTEEEEEEECSCCTTCEEEEEEEEETTEECCCEEEEECCCC TTCCCSCEEEEEEECCCCGGGC
2yeqA  57(1):APNFSSYPFTLGVASGDPLSDSVVLWTRLAPDPLN-GGGMPKQAVPVKWEVAKDEHFRKIVRKGTEMAKPSLAHSVHVEADGLEPNKVYYYRFKTGHELSPVGKTKTLPA PGANVPQMTFAFASCQQYEHG:186(130)
Rta       32:QRRWGFDPFSLGVASGSPAADSVVLWTRL-LGPGWWDSIGE-APVPVRWEVAHDQGFSRIARRGETLALAQLGHSVHAEVAGLEPDRWYFYRFTAGHAVSPTGRTRTLPA ADALPARLRLAYASCQRWEHG:160
                    **  ****** *  *********   *           *** **** ** *  *  * * *   *   * **** *  ****   * ***  **  ** * * ****   *       * ****  ***

[AVE_SECSTR] CCHHHHHHhhSCCSEEEECSCSSCCCCstsScsttsCsSCCSSSSCCSHHHHHHHHHHHHTCHHHHHHHHHSEEEECCCSTTTSTTCBTTBCSTTCsssssCHHHHHHHHHHHHHHHHHHSCCCGGGsCccs
2yeqA        YYTAYKHMAKEKLDLVFHLGDYIYEYGPNEYVSKTGNVRTHNSAEIITLQDYRNRHAQYRSDANLKAAHAAFPWVVTWDDHEVENNYANKIPEKGQ-----SVEAFVLRRAAAYQAYYEHMPLRISSLPN--:310(254)
Rta          YYGAYRHMREEQLDLVMFLGDYIYEYPN-AT-A--AVRDFPTLGWVHTLQEYRERHALHRGDVHLQAMHAACPWLVSWDDHEVQNDYAAGQAGDGRPLGLNASSDFAARRAAAYQAYYEHMPLRASALGGAL:288
             ** ** **  * ****  ********                     *** ** ***  * *  * * ** ** * ****** * **      *          *  ************** * *

[AVE_SECSTR] ssccctssCCCCEEEEETTTEEEEECCSSSSCCCCttsSSscccssssssCCHHHHCTTCCSSCHHHHHHHHHHHHCCSSEEEEECSSCCSCCCSSCSSSSCCttTTSGGGHHHHHHHHHHHHHHhTCCCEE
2yeqA        -----GPDMQLYRHFTYGNLASFNVLDTRQYRDDQANNDGN-------KPPSDESRNPNRTLLGKEQEQWLFNNLGSSTAHWNVLAQQIFFAKWNFGTSASPIYSMDSWDGYPAQRERVINFIKSKNLNNVV:431(375)
Rta          AGVAGAADVRLYGRWRFGRLADLLLLDTRQYRDRQVCGPDFKPSGQVHPSACPAWEDPGRSLLGAAQEQWLDTAFAQAGSGWTVVGQQTLFGRRDNHPGAGEQFWNDGWDGYGAARRRVTGSLQRHQVVNPV:420
                    *  **     * **    ******** *                      * * ***  *****           * *  **  *        *      ****** * * **          * *

[AVE_SECSTR] EEECSSSSEEEEEEESSTTcssSCEEEEEEEcCCSSTTCSCcScCsthhhcsshCTTEEEEEcscEEEEEEEEcsseEEEEEEEEESCSSSSCCCCEEEEEEEEEECSSTTCcEEceecccttccsbcsccthhh
2yeqA        VLTGDVHASWASNLHVDFEKTSSKIFGAEFVGTSITSGGNGADKRADTDQILKENPHIQFFN-DYRGYVRCTVTPHQWKADYRVMPFVTEPGAAISTRASFVYQKDQTGLRKVSSTTIQGGVKQSDEVEEDR:562(506)
Rta          FLGGDVHENWVGHVKADYARQGSASLGVEFCGTSITSRAGGAEQVA-Q--RLAENPHYVFADGWRRGYSVCEFTPQRLTTTLRVLDDATREDARIETQARFTVQAGRPRLERA-------------------:530
              * ****  *      *     *   * ** ******   **   *     * ****  *     ***  *  **       **    *   * * * * *  *     *

[AVE_SECSTR] hhhhhhhhhhhhhhtc
2yeqA        FFSHNKAHEKQMIKKR:(522)
Rta          ----------------
```

b

```
[AVE_SECSTR] cccccccccccCsscCSCCCCCCCssccccccsssscscsssttcbCcTTeeeeeeeeTTcbssCsttcccccttscccchhhhhtbCCSSEEEEeeEecscsssscsscssssseEEeeEEECCCCHHHHSssc
4alfA  55(8):-----------SRLLGFDSIPAATTD---------------T--ISLPKGYKSSVLISWGQP--LHKNGPAFDPSGNGTAAAQEVQFGDNNDGMSLFEFP--------GEKNRALMAINNEYTNYRYLYPHG:148(101)
Rta-1     52:------STPAARLRPGFEPVPIGAGD---------------R--LIVPKGYVASALAPWGEPVGLPGNLPVWRPDAGNTADEQAAQMGMHHDGLHYFPLD---------GSRRGLLVMNHEYVDDGLLHADG:151
Rta-2     51:---------GTGPLLGFRSVAVSTED---------------R--VTVPQGYVAQALAAWGEPVGLPGRLPPFRPDASNTAAEQEAQLGMHHDGMHFFALE---------GGRGGLLAMNHEYTDDGLLHPDG:147
Sme       77:--------EGEASPFDFKEIEAGVDE---------------T--HHVAEGYDADILLRWGDK--VFADSPEFDP-LKQTADAQSRQFGYNNDYVGFIPLE--------GNPDHGLLVVNHEYTNAEIMFPNF:172
Rta-3     68:ADVAAADVQSKALTLNFQPVPKSKAD---------------A--VLVPRGYTATPIYATGDP--LAATVPEYLNDGTDT--DFEHRAGDQHDGMEYFGLNATGTARDPNGSKRGLLVMNHEALVDQFLHANG:178
Rta-4     32:---------APAFGPGFGPLTPTLPLNTADLAVPGVFDFRDRPLISLPPGFRYWAISCTGQM--MSD---------------GTLVPGDHDGMACYRGP----------SGTTLLVRNHELNNREIKFGNA:126
                        .  ..* ...     .             .: *: . ..  .* .  .    : . ..   :.  .. . :  .*:.  . .           .. ..*:..*:*....  .....

[AVE_SECSTR] tccssssctscsssscscscsssscsccCsHHHHHHHHHHSEEEeeEEeeetttteeeecttcttceeeettseeeeestttcgggccsscssssceeeCCCSEEEEEECTTSCEEEEECSggggCbcsssssc
4alfA        ---------G------MP---------QSAEDVRKALACEGVSVIEVQRKN-GQWQFVQGSRYNRRIHGNSPLRISGPAAGHELMKTSADKHGKKVLGTFQNCANGKTPWGTYLTCEENFTDCFGSSNA-QQ:254(207)
Rta-1        ---------L------KT---------WTLEKVRKSQAAHGIAVIEVEQAAGGEWRMVRPSRFARRITANTPFNLGGPAAGHPLMRTAADPQGRTVLGTFNNCAAGITPWGTYLSGEENFRNYFAAGS----:255
Rta-2        ---------F------RT---------WNAEKVRKSQAAHGVSVIEVRQQPDGGWQVVRPSRWARRITARTPMAFGGPAAGHALLRTAADPAGLRPLGTLNNCGSGRTPWGTYLTCEENFAFYFRGPD----:251
Sme          -ARVEKVTED------GKEEDKVVLGEYTKELVDIEMAAHGGTVIEIRKVD-GKWQPVLDGKYNRRITATTEMQLSGPAAGHERVKTPSDPTGRKVFGTINNCAGGVTAWGTYMMAEENFNGYFGGELAEDH:296
Rta-3        ---------VTAATATAG---------RPVAEVDKEIAAHGVSVAEVQRRG-QSFAYDRGSRYNRRVTPLTPMQIHGPARGDGLMVTRYSPDGTTCRGTINNCGTGYTPWGTFLTGEENWAGYFTRSGG-DD:290
Rta-4        R--------G------VD---------VDPGFKYDTYANGGTSTLVLGED---GGLL-------RHYA--S-L-----------------------GTNNNCAGGPTPWGSWLSCEETTAVPNA-------:192
                                      . :..  *..*..:.:.      . .. .. ....*:... ...   ::.:. .. : ... :  ..** :**. * *:**:.:..*:.. .:

[AVE_SECSTR] ccsssscccchhhhhhtccsssscssscccttsstcttgggbtttccssssccctgggggsSsEEEECttcttsCCCEEEGGGCCSCEEEEEEEecttseseEEEEEECSeeeCEEEEEEeeeccsscgcss
4alfA        Q--------FDPAQKRYGVSAAS-REINWHPF---D--PRFDMAK--------NPNELNRHGWVVEIDPFDPQ-STPVKRTALGRFKHENAALAETDDGR-AVVYMGDDERGEFIYKFVSRDKINHRNA---:359(312)
Rta-1        S--------LDAHQRRWGLTAT-AFFRWAEH---D--PRFDAVG--------NPNEPNRFGWVVEVDPMDPA-STPVKRTALGRAAHEGAWVAVTRDGR-AVVYSGEDARNEYIYKFVSRDRIRPAGN---:359
Rta-2        R--------PGGHEARWGIGPTG-GWFRWHEH---D--ERFDARR--------HPNEPNRFGWVVEVDPFDPA-STPVKRTALGRAAHEGATVAVTRDGR-AVVYSGEDARFEYLYKFVSRDRIRPGGR---:356
Sme          P--------EFKQMKRLGTP-GG--QYEWSKF---Y--DRFDVSK--------EPNEANRFGWVVEVDPLNPT-SVPKKRTAIGRFKHEGCESIVNRDGR-VVLYSGDDERYDYVYKFVTKGTYNPDDR---:399
Rta-3        AARGGPMAKSVVSLRRSGRNAGSASRHRWETAGTDDKYARWNISQVGAGPAEDYRNELNTYGYIVEIDPYDKN-VAIRKRTALGRFAHESAAFGLRAPGQPLAVYMGDDSQGEYMYKFVSKMPWNPADAEPA:421
Rta-4        ----------------------------G---------LT--------------TYQKRHGYVFEVPALASGPTQAIPITGMGRFSHEAAAVDPA---T-GIVYLTEDTSDSLFYRYVPYQR---------:259
                                 : :  .      .:       .    :..          .:: ::.*.::*.:: ..   ....::*:.**..*.:.........:.  ..:*.:.*  .  .. *::*.......

[AVE_SECSTR] ttccssssscgggttGGGSEEEEEEEEEcsssscccsscccccsssscescEEEEECCTTsttcsttsttcCcshHHHHHTHHHHHHHHTCCCeeCEEEEEecttttccCEEEEECCbttttcssssssssscccbttbc
4alfA        --------KANRDILDHGTLYVARFDAGD--------GNPDHPKGQGQWIELTHGKNGIDAS-SG-FADQAEVLIHARLAASVVGATRMDRPEWIVVSPKDG-QVYCTLTNNAKRGE-D----GQPVGGPNP:467(420)
Rta-1        ---GLTAAQANRELLDHGTLYVARFDAD----------------GVGQWLPLVHGQGPLTAA-NG-FADQGEIVIKSRQASDLLGATKMDRPEWLAIDPVN-GTVYCTLTNNADRGKEG----MPGPDAANP:465
Rta-2        --------RGNAGLLDHGTLYAARFGAD----------------GSGRWLPLVHGQGPLTAA-NG-FADQGEVLVKTRQASDRLGATRMDRPEWIAVAADG--WVYCTLTNNSARGSVG----QPPADAANP:456
Sme          --------AANMDLFDEGTLYVAKFDED----------------GTVTWMPLVHGEGPLTAE-NG-FASQADVLIDTRLAADALGATKMDRPEDVQPNPKTG-KVYVMLTNNTKRKE-------DEIDAANP:497
Rta-3        NRI-----TTGDKYLDEGKLYVAKFAGD----------------GTGEWIELSMANPAVAGYAAYAFANQADVVVNARLAADAVGATRMDRPEWCAVHPVTG-EVYYTLTNNANRKV-SPTGSQQPVPDPANP:530
Rta-4        -----------GVLLAGGRLQALRLRDFASANTGIEFLDKLNQPMAVEWVDIPNPD--P-------V--DNLFVNSTRAQGQAAGAARFVRGEGAWWG--NG-QIYFCCTSG-GN-----------------:348
                     ..  .::.*.*:.:.:...                :  .  *  .:..  .....  .. ::..... .*.:....**:.::**:*.  . .  .    :*..:*:.  :.         . ...::

[AVE_SECSTR] cscccccsssccccccsbsSCEEEEEeecsttgggcttcseeeEEEEEECCctttsttsgsccsccttcctttCCcCEEEEEECtTtccEEEEECssccccttssgggttccSCEEEEEctttCcscccccccsss
4alfA        RE------------KNVYGQILRWRT----DRDDHASKTFAWDLFVVAGNPSVHAGTPKGG-SSNITPQNMFNSPDGLGFDKA-GRLWILTD--GDSS--NAGDFAGMGNNQMLCADPATG-----------:566(519)
Rta-1        RA------------NNAHGHIIRWREGGEDG-GDFDAARFRWDHLVLAGDPAQP----RAG-AKGNIRGDLFACPDGLAFDAR-GMLWIQTD--AHATQMYKGEFASIGNNQMLACDVATG-----------:565
Rta-2        RA------------GNAMGHILRWQE----A-GDFDGLRFRWNHFVLAGDPANP----RAE-ARGNVRGDAFACPDGLWADAR-GVLWIQTDMSAAVM--GRGEFARLGHNAMLAADPRTG-----------:552
Sme          RA------------KNAFGHIVEISE----TDGDFASTKSRWDVLLKCGDPSVAE-V-GASFSTATTRNGWFGMPDNCAIDAE-GRLWVSTD--GNNE--KDTG-RTDGVWAVETEGEGRG-----------:594
Rta-3        RSYNDAATEAPASARNVNGHIIRLRE----DGGRGAATRFAWDIYLFGAEADADP-A-RVN-LSGLNDDQDFSSPDGLWFSPKTGLCFIQTD--DG-------AYTDETNCMMLIGRPGRVGDGQPVTLNYT:646
Rta-4        --------------IGAGQVWAYDP----ARD-------TLALVVESTD----------------R--GVLEAPDNITLGPD-GRLYMYED--GG-------------NGNNIVGVNS-DG-----------:409
             :.            :. *.: ...       ....  ...:.  . ...          ..      .   .    :  **.. ...   *.:...:*  .         .  ..  ... ..   .. .:

[AVE_SECSTR] cccsccccccscsbccccccCeEEEEECcttTCEEEEEEECttSScEEEEEEESTTssssssssccssttttcccttssssssctttccttCcsctttttttccCceeeeeeeesscccttscccccccc
4alfA        --------------------EIRRFMVGPI-GCEVTGISFSPDQKTLFVGIQHPGENG-------G-------S--------TFPEHLPNG-----------KPRSSVMAITREDGGIVGA---------:633(586)
Rta-1        --------------------EVRRFLTGPT-NSEVTGMTMTPDGRTMFINIQHPGETP-------S-------D-RSDPAEPAKFSNWPDY-------RPGGRPRSSTVVIRKVDGGIVGT---------:643
Rta-2        --------------------EVRRFLVGPP-GCEVTGATMAPDGRTMFVNIQHPGENP-------S-------ERGG-----TGRSTWPDH-------RPGGRPRSATVVIRRLDGGLIGT---------:626
Sme          --------------------TSKLFFRVPV-GAEMCGPSFNPTSDTFFLAVQHPGDAGLATYEKPA-------T--------RWPDFRDDM-----------PVRPAVVAVTRQGGGKIG----------:667
Rta-3        RADGTTAEVTTHVGAKPTAATLARFLVGPV-DCEITGVTETPDGKAIFVNVQHPGESI-------SKADVNDPA--------KYTSQWPSNAGYGLGK----RPRSATLVITRDDGALIGGNGPALRNRS:756
Rta-4        --------------------ELFRVAENVVNGSEFCGGCFSHNGRFMFVNIQSPGLTL-----------------V--------IE-GPWRKG-----------Q-R---------------------:458
                                 . .::...:. ..*..* .. :....*...*:**.        .                  . ...                   ..*........:. .:
```

Figure S1. Sequence conservation within PhoD and PhoX families. (a) Pairwise sequence alignment of *B. subtilis* and *R. tataouinensis* PhoD family proteins. (b) Multiple sequence alignment of *P. fluorescens*, *S. meliloti* and *R. tataouinensis* PhoX family proteins. The average (or consensus) secondary structure is indicated above each position of the alignment (in black uppercase when completely conserved, grey lowercase otherwise): helices (G: 3_{10} helix; H: α-helix), strands (B: residue in isolated β-bridge; E: extended strand) or loops (S: bend; T: hydrogen bonded turn; C: random coil). The level of sequence conservation is represented below each position: 100% (asterisk), at least 75% (colon) or at least 50% (period) of identical amino acids. Sequences were ordered according to their pairwise similarity. The conserved active site residues are highlighted; their positions are those identified in the template 3D structures (*Bsu* PhoD: PDB chain 2yeqA; *Pfl* PhoX: PDB chain 4alfA). Sequence boundaries are given (corresponding PDB numbering between parentheses). The Ig-like domain and the catalytic domain of the PhoD proteins are separated by a space character in the alignment. The sequence of the PhoX protein of *Sinorhizobium meliloti* (Sme) corresponds to the Uniprot accession M4MZI0.
