# Supplementary material for: In Vitro and in Silico Evidence of Phosphatase Diversity in the Biomineralizing Bacterium Ramlibacter tataouinensis
Source: Front Microbiol. 2018 Jan 11;8:2592. doi: 10.3389/fmicb.2017.02592 (PMC5768637; doi:10.3389/fmicb.2017.02592)
Supplement: Supplementary file 2 [file Image2.PDF]

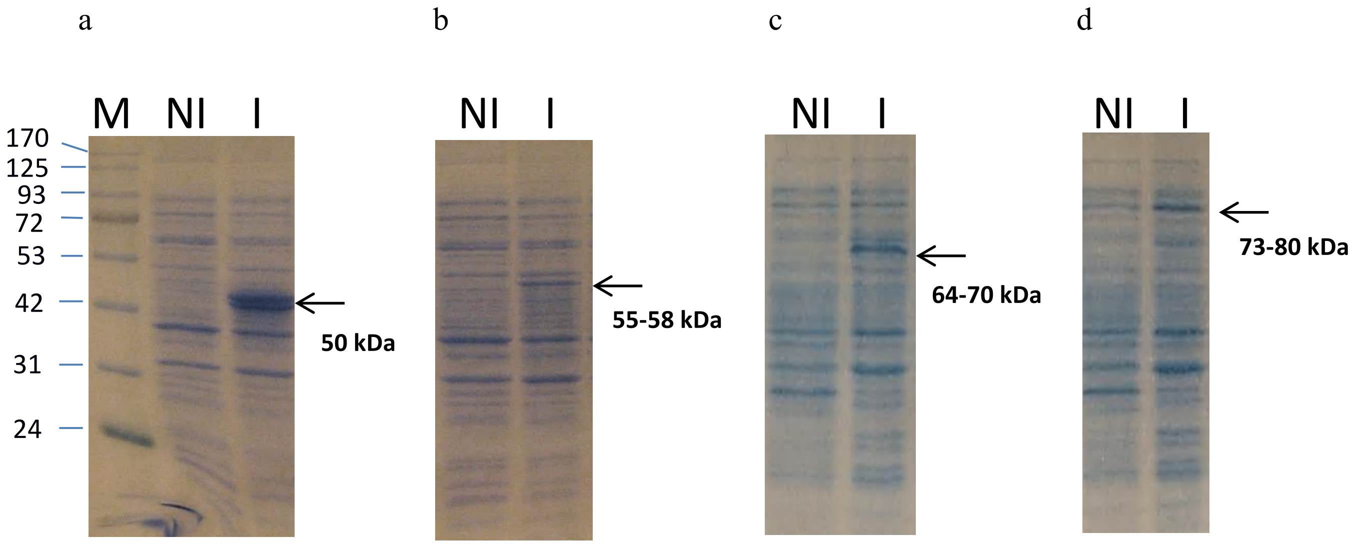

Figure S2. Heterologous expression of phosphatase genes in *E. coli*. SDS PAGE gel electrophoresis of total proteins extracted from *E. coli* clones over-expressing one of the following genes: (a) *E. coli phoA*, (b) *Rta phoD*, (c) *Rta phoX1*, (d) *Rta phoX3*. Lane M: molecular weight standards, lanes NI and I: Non-Induced and IPTG-Induced over-expression. Arrows indicate over-expressed proteins with their apparent molecular weight.
