# Supplementary material for: In Vitro and in Silico Evidence of Phosphatase Diversity in the Biomineralizing Bacterium Ramlibacter tataouinensis
Source: Front Microbiol. 2018 Jan 11;8:2592. doi: 10.3389/fmicb.2017.02592 (PMC5768637; doi:10.3389/fmicb.2017.02592)
Supplement: Supplementary file 3 [file Image3.PDF]

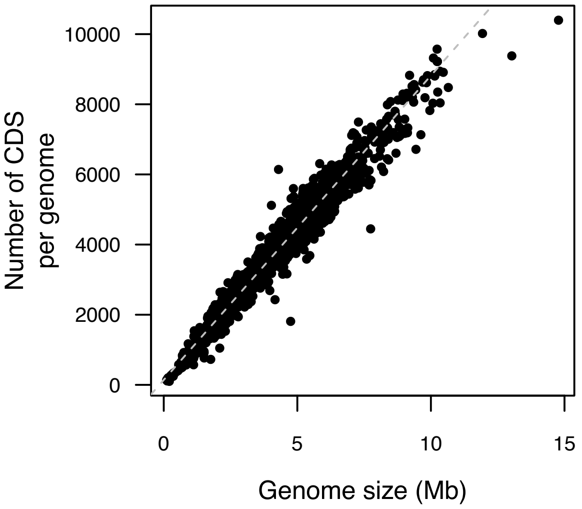

Figure S3. Linear correlation between gene content (total number of coding sequences) and genome size in our bacterial genome dataset. The dashed grey line denotes the regression line: y=867.8*x+140.9, R^2=0.97
