# Supplementary material for: In Vitro and in Silico Evidence of Phosphatase Diversity in the Biomineralizing Bacterium Ramlibacter tataouinensis
Source: Front Microbiol. 2018 Jan 11;8:2592. doi: 10.3389/fmicb.2017.02592 (PMC5768637; doi:10.3389/fmicb.2017.02592)
Supplement: Supplementary file 4 [file Image4.PDF]

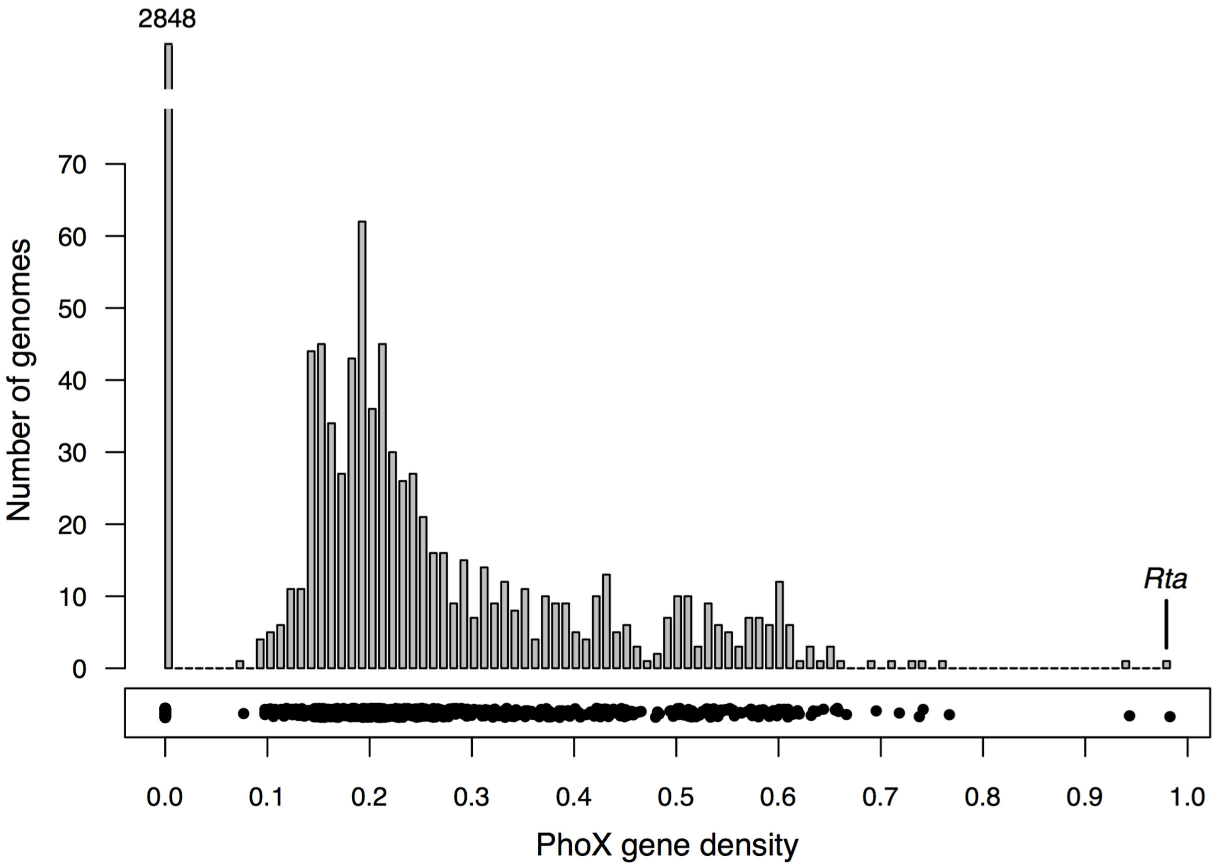

Figure S4. Distribution of PhoX gene density across bacterial genomes. Most of the genomes in our dataset do not contain any PhoX gene (the 2848 genomes with PhoX gene density strictly equal to 0 are represented by a truncated bar). *Rta* genome harbours the highest density of PhoX genes (0.983 PhoX/Mb). Six other bacteria have more than 0.69 PhoX gene per Mb (listed as follows in decreasing order according to their PhoX gene density, indicated between parentheses): *Nitrosomonas* sp. AL212 (0.943), *Corynebacterium marinum* DSM 44953 (0.767), *Methylophaga frappieri* (0.741), *Streptomyces pristinaespiralis* ATCC 25486 (0.738), *Kytococcus sedentarius* DSM 20547 (0.718), *Rubrobacter radiotolerans* (0.696).
