# Supplementary material for: In Vitro and in Silico Evidence of Phosphatase Diversity in the Biomineralizing Bacterium Ramlibacter tataouinensis
Source: Front Microbiol. 2018 Jan 11;8:2592. doi: 10.3389/fmicb.2017.02592 (PMC5768637; doi:10.3389/fmicb.2017.02592)
Supplement: Supplementary file 5 [file Table1.DOCX]

| *Time (days)* | *Medium*  *NaGP* | *Medium*  *NaGP + Ca* | *Medium*  *CaGP* | *Extracell.*  *NaGP* | *Extracell.*  *NaGP + Ca* | *Extracell.*  *CaGP* | *Cells*  *NaGP* | *Cells*  *NaGP + Ca* | *Cells*  *CaGP* |
| --- | --- | --- | --- | --- | --- | --- | --- | --- | --- |
| 0 | n.c. | 4.15 | 8.41 | n.c. | n.c. | n.c. | n.c. | 4.15 | 8.41 |
| 1 | n.c. | 4.17 | 8.46 | n.c. | 4.90 | 8.89 | n.c. | 6.39 | **10.80** |
| 3 | n.c. | 4.14 | 8.36 | n.c. | 4.89 | 8.87 | n.c. | 7.27 | **11.85** |
| 9 | n.c. | 4.06 | 8.45 | n.c. | 5.40 | 9.24 | n.c. | 8.22 | **12.60** |
| 14 | n.c. | 4.20 | 8.45 | n.c. | 5.50 | 9.27 | n.c. | 8.88 | **12.59** |
| 22 | n.c. | 4.64 | 8.57 | n.c. | 6.00 | 9.56 | n.c. | 9.07 | **12.15** |
| 29 | n.c. | 4.47 | n.c. | n.c. | 5.99 | 9.44 | n.c. | 9.16 | **11.41** |
| 35 | n.c. | 4.66 | 8.48 | n.c. | 6.27 | 9.85 | n.c. | 9.39 | **11.39** |

**Table S1. Precipitation of Ca-phosphate: saturation index (SI) of the solutions with respect to hydroxyapatite.** Bold: SI > 10 (conditions when Ca-phosphate precipitation occurs). SI was not calculated (n.c.) when concentrations of calcium or orthophosphates were below the detection limits.
