# Supplementary material for: In Vitro and in Silico Evidence of Phosphatase Diversity in the Biomineralizing Bacterium Ramlibacter tataouinensis
Source: Front Microbiol. 2018 Jan 11;8:2592. doi: 10.3389/fmicb.2017.02592 (PMC5768637; doi:10.3389/fmicb.2017.02592)
Supplement: Supplementary file 6 [file Table2.DOCX]

| ***Rta* phosphatases** | | **Blastp hits** | | | | **RPS-Blast hits** | | | |
| --- | --- | --- | --- | --- | --- | --- | --- | --- | --- |
| **Family** | **Protein sequence accession** | **Reference sequence accession** | **E-value, %identity** | **Reference sequence coverage** | ***Rta* sequence coverage** | **Reference profile accession** | **E-value** | ***Rta* sequence coverage** | **Reference profile coverage** |
| PhoD | F5XVP7 (530) | P42251  (583) | 2e-125, 45% | 82%  [64-543] | 93%  [39-530] | *COG3540*  (522) | 1e-123 | 98%  [9-530] | 99%  [5-521] |
|  |  |  |  |  |  | cd07389  (242) | 9e-48 | 58% [148-457] | 100%  [1-242] |
| PhoX | F5Y472 (643) | Q3K5N8  (633) | 0.0, 48% | 93%  [46-632] | 93%  [47-642] | *COG3211*  (616) | 1e-176 | 98%  [11-642] | 99%  [4-615] |
|  | F5Y2Z6 (626) | Q3K5N8 | 0.0, 50% | 91%  [57-632] | 91%  [55-625] | *COG3211* | 1e-179 | 99%  [4-625] | 99%  [4-615] |
|  | F5Y248 (756) | Q3K5N8 | 6e-108, 36% | 97%  [18-632] | 93%  [42-746] | *COG3211* | 1e-135 | 96%  [22-747] | 99%  [5-616] |
|  | F5Y3B1 (458) | Q3K5N8 | 2e-08, 27% | 22%  [214-353] | 22%  [158-259] | *COG3211* | 1e-67 | 99%  [1-456] | 94%  [19-595] |

**Table S2. Functional annotation of phosphatases in the genome of *Ramlibacter tataouinensis*.** Significant hits found in the predicted proteome of *Rta* by blastp and RPS-Blast against reference phosphatase sequences and profiles, respectively. For each unique sequence accession, the corresponding sequence length (in amino acids) is given between parentheses. Multi-domain profiles are indicated in italic. For each unique profile accession, the corresponding profile length (number of positions) is given between parentheses. The hit coverage on sequences and profiles are described in percent of their total length (boundary positions between brackets). The 4 *Rta* proteins identified as PhoX family members are further described as PhoX1-4 according to their order of appearance in this table.
