# Supplementary material for: In Vitro and in Silico Evidence of Phosphatase Diversity in the Biomineralizing Bacterium Ramlibacter tataouinensis
Source: Front Microbiol. 2018 Jan 11;8:2592. doi: 10.3389/fmicb.2017.02592 (PMC5768637; doi:10.3389/fmicb.2017.02592)
Supplement: Supplementary file 7 [file Table3.DOCX]

| **Query TF (*E. coli* K-12)** | | **Blastp hits** | | | |
| --- | --- | --- | --- | --- | --- |
| **Name** | **Sequence accession** | ***Rta* sequence accession** | **E-value, %identity** | **Query sequence coverage** | ***Rta* sequence coverage** |
| PhoB | P0AFJ5 (229) | F5Y146 (236) | 6e-87, 54% | 98% [4-228] | 94% [6-228] |
| Fur | P0A9A9 (148) | F5Y412 (146) | 4e-49, 52% | 91% [4-138] | 92% [3-137] |
| FadR | P0A8V6 (239) | F5XYZ9 (249) | 1e-09, 27% (27%) | 82% [20-216] (90% [25-239]) | 82% [23-227] (89% [28-248]) |
| Cra | P0ACP1 (334) | F5XVL7 (352) | 1e-13, 31% (28%) | 44% [3-149] (79% [5-269]) | 41% [22-165] (68% [24-261]) |
| FhlA | P19323 (692) | F5Y006 (470) | 3e-75, 47% (39%) | 43% [380-675] (60% [261-675]) | 62% [140-431] (83% [42-431]) |

**Table S3. Transcription factors potentially involved in the regulation of the expression of *Rta* phosphatase genes.** List of *Rta* homologs of 5 known TF (*E. coli* K-12) identified by the best blastp hits in term of E-value. The sequence accessions refer to Uniprot database. For each accession, the corresponding sequence length (in amino acids) is given between parentheses. The hit coverage on sequences are described in percent of their total length (boundary positions between brackets). In case of partial hit coverage (<90% of both the query and the Rta sequence length), the parameters of larger hit obtained with the EMBOSS Water implementation of the Smith-Waterman algorithm (exact local alignment) are indicated between parentheses.
